# Genetic Toolbox Expansion Enables Constitutively Fluorescent *Lacticaseibacillus rhamnosus* for Functional Microbiome Research

**DOI:** 10.1111/1751-7915.70405

**Published:** 2026-07-09

**Authors:** Ilke Van Tente, Marc Blanch‐Asensio, Tom Eilers, Dieter Vandenheuvel, Sarah Lebeer, Shrikrishnan Sankaran, Irina Spacova

**Affiliations:** ^1^ Laboratory of Applied Microbiology and Biotechnology, Department of Bioscience Engineering University of Antwerp Antwerp Belgium; ^2^ Bioprogrammable Materials INM‐Leibniz Institute for New Materials Saarbrücken Germany; ^3^ Saarland University Saarbrücken Germany; ^4^ U‐MaMi Centre of Excellence University of Antwerp Antwerp Belgium

## Abstract

*Lacticaseibacillus rhamnosus* strains are widely recognized for their probiotic potential and relevance in urogenital and gut health. However, their genetic tractability and genetic tools remain limited, hindering functional microbiome research and synthetic biology applications. In this study, we expanded the genetic toolbox for the widely used probiotic strains, *L. rhamnosus* GR‐1 and *L. rhamnosus* GG, by implementing direct plasmid cloning and testing of a set of genetic elements earlier validated in *Lactiplantibacillus plantarum*. Among five constitutive promoters (P_tlpA_, P_tec_, P_cpg_, P_48_ and P_23_), P_tlpA_ showed strong promoter activity in *L. rhamnosus* GR‐1. We further characterized this promoter's functionality by incorporating a repressor and assessing its native thermo‐responsiveness and stability over time, enhancing its potential for industrial applications. Using these tools, we engineered *L. rhamnosus* GR‐1 with constitutive fluorescence of mCherry, mScarlet3 and sfGFP. The functionality of these fluorescent *L. rhamnosus* GR‐1 strains was shown in a proof‐of‐concept growth competition experiment with a fluorescent pathogenic *Staphylococcus aureus* strain. These constitutively fluorescent *L. rhamnosus* strains, along with the expanded genetic toolkit, offer valuable resources for studying functional properties, such as adhesion, microbe–microbe and host–microbe interactions, and advancing *Lactobacillaceae* as a chassis for synthetic biology.

## Graphical abstract

We expanded the genetic toolkit for *Lacticaseibacillus rhamnosus* by enabling direct cloning, and leveraging five constitutive promoters and a repressor system. Fluorescent *L. rhamnosus* strains with different fluorochromes engineered with the high‐performing P_tlpA_ promoter demonstrated functionality in microbial competition assays, supporting microbiome and synthetic biology research in *Lactobacillaceae*.

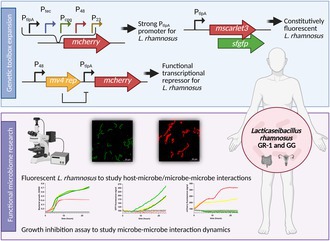

## 1 Introduction

*Lactobacillaceae*, including strains of *Lacticaseibacillus rhamnosus* species, are widely studied for their probiotic effects within various human body sites such as the vagina (Lebeer et al. 2023; Ravel et al. 2011), skin (Delanghe et al. 2021, 2024), respiratory tract (De Boeck et al. 2020; Du et al. 2022), and gastrointestinal tract (Ghosh et al. 2020; Huang et al. 2022). Among them, *L. rhamnosus* GR‐1 is a prominent urogenital probiotic with potential benefits for vaginal (Petrova et al. 2021) and gut health (Gu et al. 2022; Westerik et al. 2018). Health‐promoting properties of *L. rhamnosus* GR‐1 have been demonstrated in human clinical trials with participant numbers ranging from 110 to 139 (pregnant) women for reducing group B *Streptococcus* colonization (Ho et al. 2016; Sharpe et al. 2021) and reducing bacterial vaginosis (Anukam et al. 2006), often in combination with *Limosilactobacillus reuteri* RC‐14. Although *L. rhamnosus* GR‐1 was originally isolated from the urogenital tract, it has also shown to have benefits beyond its isolation site. For example, it has shown anti‐allergic effects following nasal and oral administration in a murine asthma model (Spacova, Van Beeck, et al. 2020), promoted cardiovascular health in a rat model (Gan et al. 2014), and even supported bee health in a field trial (Daisley et al. 2020, 2023). Another closely related strain, *L. rhamnosus* GG, was originally isolated from faeces from a healthy adult and is currently one of the most studied probiotics for human health (Capurso 2019; Mathipa‐Mdakane and Thantsha 2022; Rohani et al. 2016; Yan and Polk 2012), demonstrated in multiple human clinical trials in adults and children (Damholt et al. 2022; Groah et al. 2019; Schnadower et al. 2018). For example, intake of *L. rhamnosus* GG capsules led to a reduction in endotoxemia and microbiome dysbiosis in 30 cirrhosis patients in the USA (Bajaj et al. 2014). In addition, potential antiviral activity of *L. rhamnosus GG* was observed when applied in a multi‐strain throat spray in 78 COVID‐19 outpatients in Belgium (De Boeck et al. 2022).

Building further on their probiotic potential, *L. rhamnosus* represents a promising candidate for functional microbiome research further unravelling probiotic mechanisms, as well as for application as a live biotherapeutic product or chassis in synthetic biology (Mugwanda et al. 2023). Currently, widely used model microorganisms such as *Escherichia coli* and *Saccharomyces cerevisiae* are typically selected as the chassis of choice for applications involving genetic engineering due to their well‐established and robust genetic toolboxes. However, their application in human‐associated and clinical settings remains limited by safety concerns and reduced ecological relevance (Lee et al. 2008; Nielsen 2019). In contrast, probiotic *L. rhamnosus* strains are excellent alternatives in the context of human health applications due to their safety and multifactorial beneficial action (Mathipa‐Mdakane and Thantsha 2022; Petrova et al. 2021). However, their genetic toolbox—including the availability of strong constitutive promoters—remains limited (Mathipa‐Mdakane and Thantsha 2022). Expanding these tools would enable deeper mechanistic research and support the development of *L. rhamnosus* as a safe and effective therapeutic platform.

To date, most of the attempts in expanding the genetic toolbox of *Lactobacillaceae* have focused on species other than *L. rhamnosus* , especially *Lactiplantibacillus plantarum* (Blanch‐Asensio, Dey, and Sankaran 2023; Blanch‐Asensio, Dey, Tadimarri, and Sankaran 2023; Dey et al. 2023; Mugwanda et al. 2023; Wiull et al. 2025) and *Limosilactobacillus reuteri* (van Pijkeren and Barrangou 2017). However, it is also to be noted that the discovery of novel, well‐performing genetic parts in one specific species might exhibit different activity in other *Lactobacillaceae* species. One example is the pSIP system, an inducible gene expression system that works in multiple *Lactobacillaceae* species, including *Lactiplantibacillus pentosus*, *Latilactobacillus sakei* and *L. plantarum* (Karlskås et al. 2014; Wiull et al. 2024) but is not functional in *Lactobacillus helveticus* and *Lactobacillus johnsonii* (Karlskås et al. 2014). Indeed, this expression system cannot be applied universally across *Lactobacillaceae* species due to host specificity of plasmid replicons, strain‐specific promoter variability, and the requirement for a specific transport protein to facilitate uptake of the SppIP inducer peptide (Karlskås et al. 2014). However, cross‐species tool testing can be an interesting strategy to expand genetic engineering toolboxes of other less‐studied *Lactobacillaceae* species such as *L. rhamnosus* (Sørvig et al. 2003).

Several attempts have been made to expand the genetic toolbox of *L. rhamnosus* for heterologous protein production (Mathipa‐Mdakane and Thantsha 2022; Spacova et al. 2018). For example, a nisin‐inducible expression system (NICE) was successfully implemented for expressing fluorescent proteins (Spacova et al. 2018), anti‐HIV lectins (Petrova et al. 2018), and even birch pollen allergens (Spacova, Van Beeck, et al. 2020) in *L. rhamnosus* GG and *L. rhamnosus* GR‐1. Nevertheless, this expression system presents limitations such as leaky expression, requirement for inducer addition and dual plasmid composition (Beltran et al. 2016; Geoffroy et al. 2000; Mierau and Kleerebezem 2005). Alternatively, several constitutive promoters have been explored in *L. rhamnosus* for production of therapeutic or fluorescent proteins, including the Major Secreted Protein 1 promoter (P_msp1_) from *L. rhamnosus* GG and the Aggregation Promoting Factor promoter (P_apF_) from *Lactobacillus crispatus* (Günaydin et al. 2014; Petrova et al. 2018). However, the expression levels reported with these promoters in *L. rhamnosus* strains remained limited. In addition to promoters, fluorescent reporters are highly useful for bacterial imaging, promoter strength evaluation and studying probiotic properties in vitro (Dean and Palmer 2014; Landete et al. 2016). Although different fluorescent reporters are available, strain‐specific functionality has been observed. For example, while mCherry and mTagBFP2 showed long‐lasting fluorescence signal in *L. rhamnosus* GR‐1, this was not the case for green fluorescent protein (GFP) and enhanced cyan fluorescent protein (ECFP) (Spacova et al. 2018). In another study, GFP under expression of P_ldhL_ in *L. rhamnosus* GG did not lead to a detectable fluorescence signal (De Keersmaecker et al. 2006). Despite the recent advances in the development of a broad range of fluorescent reporters with increased photostability, brightness and wide spectral range (Chapman et al. 2008; Gadella et al. 2023; Pédelacq et al. 2006), such as mScarlet3 and superfolder green fluorescent protein (sfGFP), the functionality of these proteins remains poorly understood in *L. rhamnosus* .

In this study, we explore the functionality of different genetic parts—including constitutive promoters, a transcriptional repressor, and fluorescent reporters—in *L. rhamnosus* GR‐1 and *L. rhamnosus* GG, aiming to expand their genetic toolboxes and develop constitutively fluorescent *L. rhamnosus* engineered strains. For that, we cloned and evaluated promoters previously tested in *L. plantarum* WCFS1 (Blanch‐Asensio et al. 2024; Dey et al. 2023; Meng et al. 2021; Rud et al. 2006) in *L. rhamnosus* , investigating the relevance of cross‐species genetic tool testing. Additionally, we tested the possibility of successfully transforming *L. rhamnosus* GR‐1 with an intermediate‐free, time‐saving, direct cloning protocol previously published in *L. plantarum* WCFS1 (Blanch‐Asensio, Dey, and Sankaran 2023). Subsequently, we demonstrated the applicability of the constitutive fluorescent *L. rhamnosus* strains for functional research in a competition assay with the opportunistic pathogen *Staphylococcus aureus* . Overall, we aimed to expand the genetic toolbox of *L. rhamnosus* and elucidate the potential of *L. rhamnosus* for unravelling probiotic mechanisms and enhancing its synthetic biology applicability.

## 2 Experimental Procedures

### 2.1 Bacterial Strains, Plasmids and Growth Conditions

*Lacticaseibacillus rhamnosus* strains were grown in the De Man Rogosa Sharpe (MRS) medium (Carl Roth) (De Man et al. 1960) at 37°C on the Unimax 1010 shaking platform (Hedolph EMEA) at 250 rpm. For recombinant *L. rhamnosus* strains constructed in this study, 10 μg/mL erythromycin (Carl Roth) was added to the medium. For growth and fluorescence induction in strain CMPG11265 (derived from *L. rhamnosus* GR‐1) and strain CMPG11261 (derived from *L. rhamnosus* GG) expressing mCherry from the inducible nisA promoter (NICE system), previously published protocols were used (De Keersmaecker et al. 2006; Spacova et al. 2018). Briefly, an overnight culture in MRS with 5 μg/mL erythromycin and 10 μg/mL chloramphenicol was diluted 1:50 in fresh medium, and after 30 min was induced with 500 ng/mL nisin from *Lactococcus lactis* (Sigma‐Aldrich) for 24 h. For thermo‐responsiveness experiments, *L. rhamnosus* GR‐1 pAMB6500 was grown at different temperatures: 30°C, 33°C, 37°C and 39°C. *Escherichia coli* DH5α strains were grown in Lysogeny broth (LB) (Carl Roth), shaking (250 rpm) at 37°C. Recombinant *E. coli* DH5α were grown in LB media supplemented with 250 μg/mL erythromycin. MRS (Difco) and LB (Carl Roth) agar plates were incubated at 37°C under static conditions. GFP‐expressing *S. aureus* NR‐51163 carrying the fluorescent reporter plasmid pSGFPS1 (obtained through BEI resources, NIAID, NIH) was grown in Brain Heart Infusion (BHI) medium (Becton Dickinson) supplemented with 10 μg/mL trimethoprim (Thermo Scientific) at 37°C under shaking (250 rpm) conditions. All bacterial strains and plasmids used in this study can be found in Table S1.

### 2.2 Construction and Confirmation of Plasmids

Plasmids were constructed using the molecular HiFi DNA Assembly Cloning Kit (New England Biolabs) (Gibson et al. 2009) and used according to the manufacturer's instructions. High‐fidelity Phusion PCR (Thermo Fisher Scientific) was used for insert and plasmid backbone amplification. PCR primers were purchased from Integrated DNA Technologies (Belgium). Primers are listed in Table S2. Assembled plasmids were transformed by heat shock into chemically competent *E. coli* DH5α (New England Biolabs) for plasmid amplification using earlier described protocols (Sambrook et al. 1989). For plasmid extraction, the commercially available Monarch Plasmid Miniprep kit (New England Biolabs) and GeneJet Plasmid Midiprep Kit (ThermoFisher Scientific) were used according to the manufacturer's instructions.

The pAMB6505 and pAMB6506 plasmids were constructed using an in vitro PCR‐based amplification method for direct cloning in *Lactobacillaceae* (Blanch‐Asensio, Dey, and Sankaran 2023). Phusion PCR was used for linearisation of the plasmids. PCR products were purified using the NucleoSpin Gel and PCR Clean‐up Kit (Macherey‐Nagel), according to the manufacturer's instructions. Enzymes for phosphorylation (Quick Blunting Kit) and ligation (T4 DNA ligase) were purchased from New England Biolabs and used following the manufacturer's instructions.

Successful transformation of *E. coli* DH5α and *L. rhamnosus* was confirmed by performing Taq polymerase PCR (Thermo Fisher Scientific) and analysing Sanger sequencing results of PCR products in duplicate due to forward and reverse reads (Neuromics Support Facility, Antwerp).

### 2.3 Production of Electrocompetent *L. rhamnosus* and Electroporation of *L. rhamnosus*

Plasmids were transformed into electrocompetent *L. rhamnosus* GR‐1 and *L. rhamnosus* GG using a similar electroporation protocol as described earlier (De Keersmaecker et al. 2006; Petrova et al. 2016). Briefly, overnight cultures of wild type *L. rhamnosus* strains were diluted in MRS medium supplemented with 2% glycine (Merck) to an optical density at 600 nm (OD_600_) of 0.1 and incubated to the exponential phase corresponding to OD_600_ of 0.3–0.4. OD_600_ was measured using a spectrophotometer (Thermo Scientific Genesys 20). During this incubation step, 3 μg/mL ampicillin (Carl Roth) was added when the OD_600_ of 0.2 was reached. After incubation, bacterial cells were centrifuged (400 rpm, 10 min at 4°C) in the Sigma 2‐16P centrifuge and washed twice with an equal volume of ice‐cold washing buffer (0.5 M sucrose [Millipore Sigma Aldrich], 7 mM KH_2_PO_4_ at pH 7.4 [Thermo Scientific] and 1 mM MgCl_2_ [Chem‐lab]) and concentrated in 1 mL washing buffer after washing. One µg of the plasmid DNA was added to 100 μL competent *L. rhamnosus* and incubated for 30 min on ice. Before electro‐pulse (1.7 kV, 25 μF, 200 Ω) with GenePulser Xcell (Bio‐Rad), this mixture was transferred to a chilled 2 mm electroporation cuvette (Bio‐Rad). After the pulse, 1 mL of preheated recovery medium (MRS supplemented with 2 mM CaCl_2_ (Chem‐lab) and 20 mM MgCl_2_) was added, followed by 3 h of shaking incubation at 37°C before plating on agar plates with appropriate antibiotics. Plates were incubated for 2 days at 37°C.

### 2.4 Assessing Promoter Strength and Fluorescence Reporter Activity in *L. rhamnosus*

Different constitutive promoters (Table 1) and fluorescent reporters (Table 2) were tested for functionality in *L. rhamnosus*. Readouts for assessing promoter and fluorescent reporter functionality in *L. rhamnosus* were performed using the Synergy HTX plate reader (BioTek, Winooski, VT, USA) for mCherry fluorescence and the Leica DMi8 fluorescence microscope for mCherry, mScarlet3 and sfGFP fluorescence.

**TABLE 1:** Characteristics of the different selected promoters for evaluation in *L. rhamnosus* .

Promoter	Origin	Description	Reference in *Lactobacillaceae*
P_tlpA_	*Salmonella enterica* subspecies *enterica* serovar Typhimurium	Responsible for thermo‐responsive gene regulation of *tlpA* together with its repressor	Dey et al. (2023)
P_tec_	Mv4 phage infecting *Lactobacillus delbrueckii*	Naturally driving expression of the Tec repressor	Blanch‐Asensio et al. (2024) and Coddeville et al. (2007)
P_cpg_	Φg1e phage infecting *L. plantarum* G1e	Naturally driving expression of the Cpg repressor	Kakikawa et al. (2000)
P_48_	Synthetic	Originating from random mutagenesis of the sequence between conserved promoter regions of the 16S rRNA promoter of *L. plantarum* WCFS1	Rud et al. (2006)
P_23_	*Lactococcus lactis*	Reported strong promoter in lactic acid bacteria	Meng et al. (2021)
P_nisA_	*Lactococcus lactis*	Strong inducible promoter for *L. rhamnosus* GR‐1 and *L. rhamnosus* GG as part of the NICE system, included in this study for comparison	Mierau and Kleerebezem (2005)

*Note:* Name, origin, description and references are included for each promoter.

**TABLE 2:** Characteristics of the different selected fluorescent proteins.

Fluorescent protein	Size (kDa)	Organism	Colour	Rationale for testing in *L. rhamnosus* GR‐1	References
mScarlet3	26.4	Synthetic	Red	Improved brightness, photostability and maturation speed compared to mCherry	Gadella et al. (2023)
mCherry	26.7	*Discosoma* sp.	Red	Commonly used reporter in *Lactobacillaceae*	Shaner et al. (2004)
sfGFP	26.8	*Aequorea victoria*	Green	Rapid folding and higher stability compared to regular GFP	Pédelacq et al. (2006)

*Note:* For each fluorescent protein, the name, size in kilodalton (kDa), origin, colour, rationale for testing in *L. rhamnosus* GR‐1 and reference are included.

For single point fluorescence measurements, recombinant *L. rhamnosus* strains inoculated from glycerol stock were grown in 10 mL MRS medium supplemented with 10 μg/mL erythromycin. After 16 h, cultures were centrifuged at 2000 × *g* for 3 min, and the supernatant was discarded. Next, the bacterial cell pellets were washed twice and resuspended in 1 mL of phosphate‐buffered saline (PBS) (8 g/L NaCl (Sigma Aldrich), 0.2 g/L KCl (Sigma Aldrich), 4.2 g/L Na_2_HPO_4_ (Sigma Aldrich) and 0.25 g/L KH_2_PO_4_ (Sigma Aldrich) adapted to pH 7.4). Two hundred μL of the mixture was added to a white 96‐well plate with transparent flat bottom (Greiner) with 3 biological and 3 technical replicates. Optical density at 600 nm (OD_600_) and bottom mCherry fluorescence measurements at excitation (Ex) (nm): 580/50 and emission (Em) (nm): 635/32 wavelengths were measured and recorded. Relative fluorescence units were calculated by normalizing fluorescence values for bacterial growth (OD_600_).

To follow up fluorescence and bacterial growth over time, a 24 h run was performed with the Synergy HTX plate reader. Overnight bacterial cultures were diluted to OD_600_ of 0.2 in fresh medium with appropriate antibiotics in a transparent 96‐well plate with lid (VWR). Readout was performed at 37°C under static conditions and every 15 min mCherry fluorescence (Ex: 580/50 nm, Em: 635/32 nm) and OD_600_ were recorded. Before every measurement, the plate was orbitally shaken for 10 s.

For visualization of fluorescent lactobacilli using fluorescence microscopy, 1 mL of overnight cultures of recombinant *L. rhamnosus* GR‐1 were centrifuged for 3 min at 2000 × *g* and washed with 1 mL of PBS. After that, 3 μL of the bacterial suspension was applied on a glass microscopy slide (VWR) and covered by a glass cover slide with a thickness of 1.5 (VWR). The bacteria were imaged at 40‐fold magnification using phase contrast microscopy for visualization of the bacteria, the TexasRed filter for visualization of red fluorescent proteins, and the FITC filter for visualization of green fluorescent proteins.

### 2.5 Assessing Longitudinal mCherry Production Stability Over Time and Plasmid Retention Based on Antibiotic Resistance

Longitudinal mCherry production and plasmid stability based on antibiotic resistance of *L. rhamnosus* GR‐1 carrying the pAMB6500 plasmid were evaluated in a subculture experiment over three consecutive days. Following an initial glycerol stock subculture step, overnight bacterial cultures (10 mL) were diluted 1:50 in fresh MRS medium either supplemented with 10 μg/mL erythromycin (selective conditions) or without antibiotics (non‐selective conditions) and incubated for 24 h. This subculturing step was repeated daily for 3 days. At each time point (every 24 h), OD_600_ and mCherry fluorescence were measured using a Synergy HTX plate reader to monitor mCherry production stability over time.

In parallel, plasmid retention was assessed under non‐selective conditions. Cultures were serially passaged (1:50) every 24 h in antibiotic‐free medium. At each time point (every 24 h), cultures were serially diluted and plated on agar plates with and without erythromycin (10 μg/mL). After 48 h incubation, colony‐forming units (CFUs) were enumerated, and plasmid retention was calculated as the ratio of CFUs on selective versus non‐selective plates. These values were expressed as percentages, representing the proportion of cells retaining the plasmid over time.

### 2.6 Time‐Course Growth Competition Experiment of *L. rhamnosus* GR‐1 pAMB6500 With Pathogenic Fluorescent *S. aureus* NR‐51163

The protocol was adapted from previous work (Spacova, O'Neill, and Lebeer 2020; van den Broek et al. 2018). Overnight cultures of GFP‐expressing *S. aureus* NR‐51163 and (recombinant) *L. rhamnosus* GR‐1 strains were prepared in 10 mL BHI and 10 mL MRS media supplemented with, respectively, 10 μg/mL trimethoprim and 10 μg/mL erythromycin (no antibiotics were added for wild type *L. rhamnosus* GR‐1), and incubated shaking (250 rpm) at 37°C. OD_600_ of overnight cultures were measured, cultures were centrifuged (10 min at 4000 × *g*) and bacterial cell pellets were washed twice with 1× PBS to remove remaining antibiotics. After that, *L. rhamnosus* GR‐1 strains or *S. aureus* NR‐51163 were resuspended in MRS (Carl Roth) or BHI medium, respectively, to obtain a final concentration of 10^9^ CFU/mL for (recombinant) *L. rhamnosus* GR‐1 or 10^7^ CFU/mL for *S. aureus* NR‐51163 (stock solutions). A mixture of 140 μL Muellor‐Hinton (MH) medium (HiMedia) and 60 μL BHI medium (70:30 ratio) was added to wells of a transparent 96‐well plate with lid. This combination of media was used as MRS influences the growth properties of *S. aureus* NR‐51163. Glucose can be added to promote *L. rhamnosus* growth and medium acidification. Then, 2 μL of (recombinant) *L. rhamnosus* GR‐1 and/or *S. aureus* NR‐51163 stock solutions were added to each well. All conditions were tested in triplicate. As a positive control, 2 μL hextril (Johnson & Johnson), an antiseptic, was added together with *S. aureus* NR‐51163. Medium without bacteria was used as a negative control. Kinetic growth was assessed in a Synergy HTX plate reader over the course of 24 h at 37°C under static conditions. OD_600_ combined with GFP (Ex [nm]: 485/20 and Em [nm]: 528/20) and mCherry (Ex [nm]: 580/50 and Em [nm]: 635/32) fluorescence measurements were taken every 15 min after 10 s of orbital shaking.

Dilution series of stock solutions of (recombinant) *L. rhamnosus* GR‐1 and *S. aureus* NR‐51163 were plated on respectively MRS and BHI agar plates supplemented with appropriate antibiotics to check initial bacterial cell concentrations. At the end of the 24 h 96‐well plate readout, (recombinant) *L. rhamnosus* GR‐1 and *S. aureus* NR‐51163 in single culture and grown in combination were plated on selective media agar plates to estimate the CFU/mL of each strain. Combination cultures were spotted on both BHI agar plates containing 10 μg/mL trimethoprim to select for *S. aureus* NR‐51163 and MRS agar plates containing 10 μg/mL erythromycin to select for *L. rhamnosus* GR‐1 pAMB6500. For *L. rhamnosus* GR‐1, MRS agar plates without antibiotics were used.

### 2.7 Antimicrobial Spot Assay of *L. rhamnosus* GR‐1 With *S. aureus* NR‐51163

*Lacticaseibacillus rhamnosus* GR‐1 and *L. rhamnosus* GR‐1 pAMB6500 were inoculated from glycerol stocks into 10 mL MRS medium (Carl Roth) supplemented with appropriate antibiotics and cultured overnight at 37°C (shaking conditions). Three biological replicates were prepared. On the following day, 5 μL of each culture was spotted in technical triplicate onto MRS agar plates without antibiotics and incubated at 37°C for 48 h. In parallel, *S. aureus* NR‐51163 was inoculated into 10 mL BHI medium containing trimethoprim (10 μg/mL) and grown under shaking conditions for 24 h at 37°C (three biological replicates). Subsequently, 45 μL of *S. aureus* NR‐51163 culture was mixed with 20 mL per plate of soft BHI agar (5 g/L) and overlaid onto the MRS plates containing lactobacilli. Control conditions included hextril and clindamycin (Cayman Chemical) antibiotics. Plates were incubated at 37°C for 24 h after which antimicrobial activity was evaluated by measuring inhibition zones (halo formation).

### 2.8 Statistical Analyses

Statistical analyses and plotting were performed using the software GraphPad Prism version 10.5.0 (GraphPad Prism Software Inc., San Diego, CA, USA, www.graphpad.com). Prior to analysis, normality and homogeneity of variance were assessed using the Shapiro–Wilk test (*α* = 0.05) and Brown Forsythe/*F* test (*α* = 0.05). Depending on data distribution and experimental design, either ordinary one‐way ANOVA with Tukey's post hoc test or Welch's ANOVA with Dunnett T3 multiple comparison correction was applied. Non‐parametric data were analysed using the Kruskal–Wallis test with Dunn's correction. Pairwise comparisons were conducted using unpaired *t*‐tests or multiple *t*‐tests with Holm–Šídák correction (*α* = 0.05). Area under the curve (AUC) was calculated for growth competition experiments prior to statistical comparison. A significance threshold of *α* = 0.05 was used, with significance levels indicated as ns (not significant), *p‐value < 0.05, **p‐value < 0.005, ***p‐value < 0.0005 and ****p‐value < 0.00005. All experiments included three independent biological replicates and three technical replicates. Graphs display mean values, standard deviations, individual data points and statistical significance levels.

## 3 Results

### 3.1 Identification of a Strong Constitutive Promoter for *L. rhamnosus*

The identification of strong constitutive promoters is an essential parameter for establishing *L. rhamnosus* as a model chassis for *Lactobacillaceae* engineering and for elevating their functional probiotic potential. Here, we started from non‐endogenous constitutive promoters previously validated in the more genetically accessible *L. plantarum* WCFS1 (Kleerebezem et al. 2003) and evaluated their activity in *L. rhamnosus* GR‐1 and *L. rhamnosus* GG. Five promoters, originating from different sources, were evaluated for functionality in *L. rhamnosus* (Table 1): two phage‐derived promoters, namely P_cpg_ and P_tec_ (Blanch‐Asensio et al. 2024), one promoter derived from phylogenetically distant *Salmonella enterica* subsp. *enterica* serovar Typhimurium, namely P_tlpA_ (Dey et al. 2023), the strong lactococcal P_23_ promoter (Meng et al. 2021), and the synthetic promoter P_48_, which can potentially bypass promoter cross‐reactivity and improve overall transcription rates (Rud et al. 2006). Promoters like P_tlpA_ or phage‐derived promoters (P_tec_ and P_cpg_), which are tightly regulated, offer powerful alternatives to constitutive promoters such as P_32_, a strong *L. lactis* promoter involved in stress response and protein folding (Zhu et al. 2015). The NICE system containing the inducible *nisA* promoter was included for comparison, as it has previously been used for successful construction of fluorescent *L. rhamnosus* GG and *L. rhamnosus* GR‐1 (Spacova et al. 2018).

**FIGURE 1:**
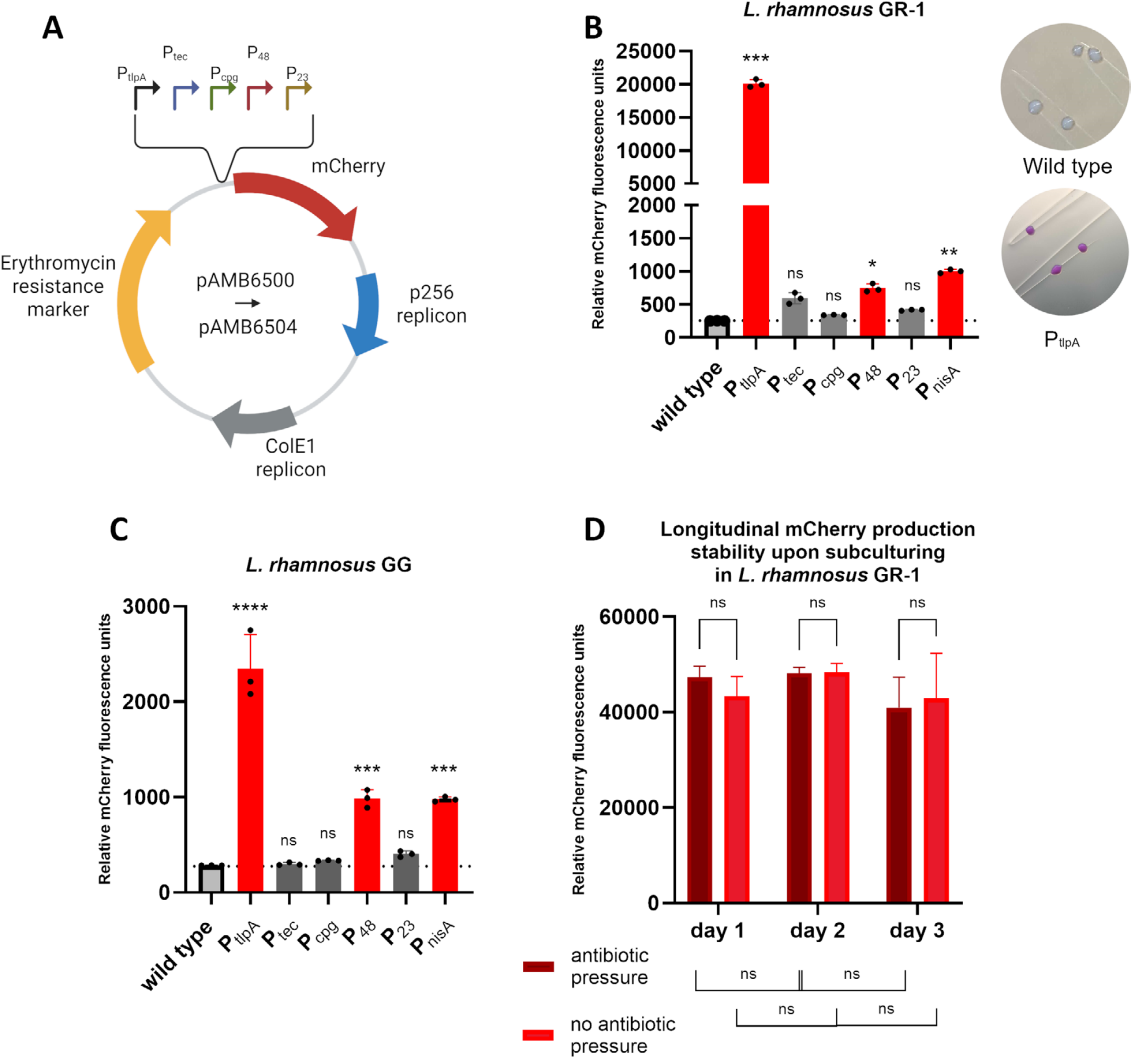
Constitutive promoter evaluation in *Lacticaseibacillus rhamnosus* GR‐1 and *L. rhamnosus* GG based on mCherry fluorescence. (A) Plasmid map for constitutive promoter evaluation in *L. rhamnosus* (also see Table S1). Different constitutive promoters (P_tlpA_, P_tec_, P_cpg_, P_48_ and P_23_) were cloned upstream of the *mCherry* gene in the narrow‐host range p256 plasmid backbone, resulting in respectively plasmids: PAMB6500 (P_tlpA_), pAMB6501 (P_tec_), pAMB6502 (P_cpg_), pAMB6503 (P_48_) and pAMB6504 (P_23_). Relative mCherry fluorescence units (fluorescence divided by OD_600_) of recombinant *L. rhamnosus* GR‐1 (B) and *L. rhamnosus* GG (C) are shown. The P_nisA_ promoter in the inducible NICE system was included for comparison of promoter activity. Photos of wild‐type *L. rhamnosus* GR‐1 and *L. rhamnosus* GR‐1 pAMB6500 colonies on agar plates are shown. (D) Longitudinal mCherry production stability was assessed in *L. rhamnosus* GR‐1 driven by P_tlpA_ in a 3‐day serial subculture experiment. mCherry fluorescence measurement of cultures of *L. rhamnosus* GR‐1 pAMB6500 were performed every 24 h after which cultures were diluted in fresh media with and without erythromycin antibiotics. Mean values with standard deviations of three independent biological replicates are depicted in the graphs. Significance testing was performed against wild type strains (for panel B and C) and significance levels are indicated on the graphs: *p‐value < 0.05, **p‐value < 0.005, ***p‐value < 0.0005, ****p‐value < 0.00005 and ns, not significant.

These non‐endogenous promoters (Table 1) were cloned upstream of the *mCherry* gene in the narrow host‐range p256 backbone (Sørvig et al. 2005) (Figure 1A). p256 contains a low‐copy origin of replication, with copy number established at three to 10 copies in closely related lactobacilli (Sørvig et al. 2005; Tauer et al. 2014). This plasmid backbone could be transformed with sufficient electroporation efficiency of > 10^3^ CFU/μg in *L. rhamnosus* and was therefore used as a screening vector for the different constitutive promoters evaluated in this study.

The plate reader assay based on relative mCherry fluorescence showed that different promoters, namely the constitutive P_tlpA_ and P_48_ and P_nisA_ in the inducible NICE system, showed activity (*p* < 0.05) in *L. rhamnosus* GR‐1 (Figure 1B) and *L. rhamnosus* GG (Figure 1C). Activity differed between promoters and between bacterial strains, indicating variation in promoter strength. No mCherry signal could be detected for P_tec_, P_cpg_ and P_23_ in both *L. rhamnosus* strains. Among the tested promoters, P_tlpA_ led to the highest levels of mCherry fluorescence in *L. rhamnosus* GR‐1 and *L. rhamnosus* GG. However, promoter activity of P_tlpA_ was 8.6‐fold higher in *L. rhamnosus* GR‐1 compared to *L. rhamnosus* GG. In *L. rhamnosus* GR‐1, 20‐fold higher activity was observed for P_tlpA_ compared to the previously reported P_nisA_ in the NICE system serving as reference for comparison. P_48_ activity was comparable for both *L. rhamnosus* strains. Comparison of promoter activity between *L. rhamnosus* GR‐1 and *L. rhamnosus* GG is depicted in Figure S1. Fold changes of the different promoters compared to the wild type are presented in Table S3 for *L. rhamnosus* GR‐1 and in Table S4 for *L. rhamnosus* GG. Given the strong and more pronounced activity of P_tlpA_ in *L. rhamnosus* GR‐1, we have chosen to further characterize this promoter. The strong promoter activity of P_tlpA_ in *L. rhamnosus* GR‐1 is also clear from the red coloration of the colonies on plate (Figure 1B).

To assess the industrial applicability of this strain, the longitudinal mCherry production stability in *L. rhamnosus* GR‐1 by P_tlpA_ was assessed over time in a 3 day serial subculture experiment (Figure 1D). P_tlpA_ exhibited consistent mCherry production without significant changes over 3 days both with and without antibiotic selection pressure, and stronger activity following serial subculturing compared to the initial fluorescent values after overnight growth was observed (~20,000 and ~40,000 RFUs, respectively). These results suggest that P_tlpA_ is a highly active and longitudinally stable promoter in *L. rhamnosus* GR‐1 and may be well suited for long‐term industrial applications. In addition, pAMB6500 plasmid retention based on antibiotic resistance was confirmed for 3 days for *L. rhamnosus* GR‐1 (no significant changes in CFU/mL counts on plates with vs. without antibiotics) (Figure S2).

To further assess the potential functionality of the P_tlpA_ promoter in *L. rhamnosus* GR‐1 in conditions equivalent to living systems or industrial bioreactors, the temperature‐dependent activity of the P_tlpA_ promoter was evaluated. For that, *L. rhamnosus* GR‐1 pAMB6500 cultures were grown at different temperatures (30°C, 33°C, 37°C and 39°C) and levels of mCherry fluorescence were measured using the plate reader (Figure S3). The strongest fluorescence level due to P_tlpA_ activity was observed at 37°C, while significantly lower fluorescence values were recorded for lower (30°C [*p* < 0.005] and 33°C [*p* < 0.005]) and higher (39°C [*p* < 0.05]) temperatures. Compared to the wild‐type background, significantly higher mCherry fluorescence was measured for all tested temperatures (*p* < 0.00005).

### 3.2 Repressing the Expression Levels of P_tlpA_ Promoter in *L. rhamnosus* GR‐1

**FIGURE 2:**
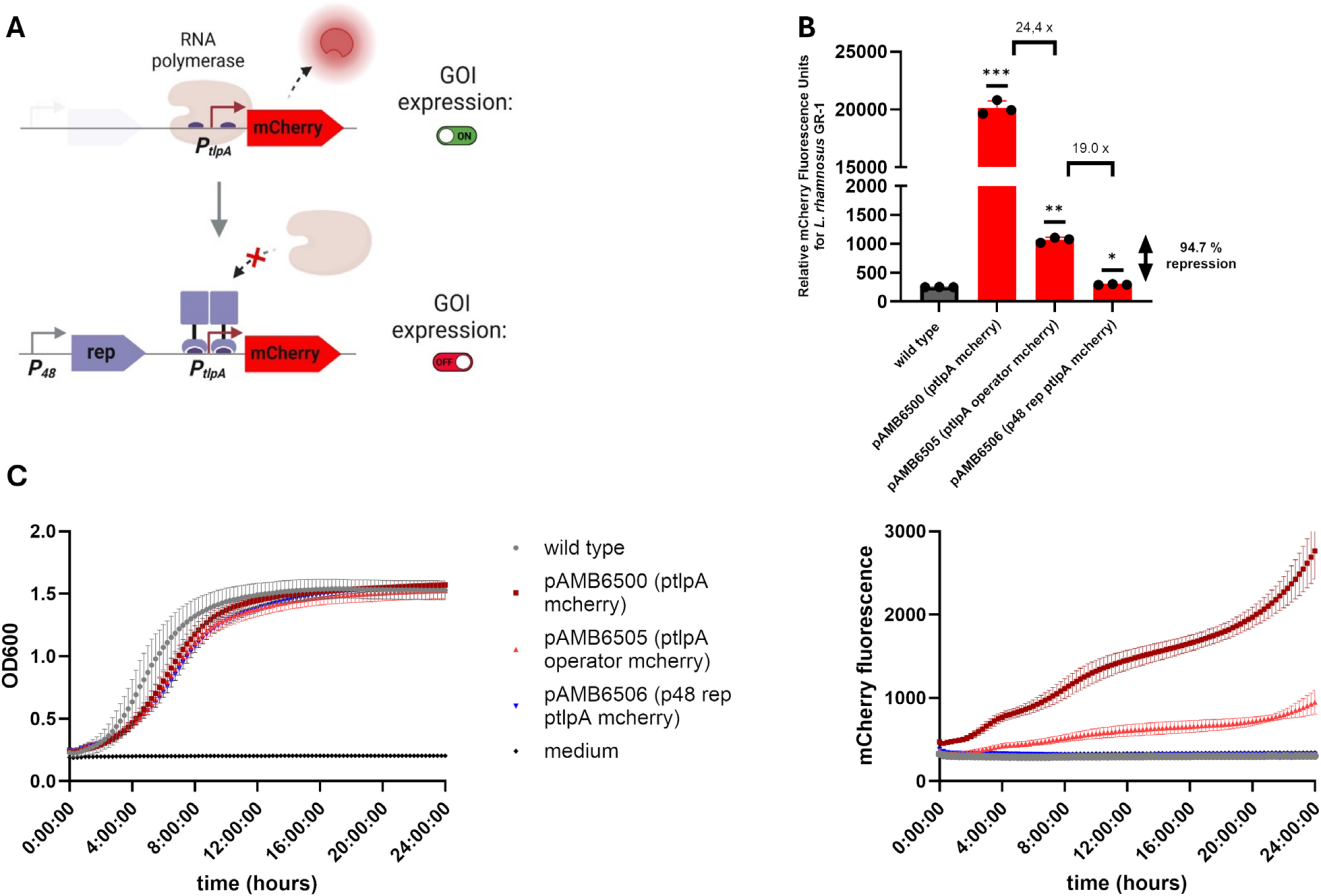
Assessing functionality of a repressor‐promoter system based on P_tlpA_ in *Lacticaseibacillus rhamnosus* GR‐1. (A) Design of the full promoter‐repressor construct. P_48_ regulates the transcription of the Rep repressor. Binding of the Rep repressor at the operator sites flanking the P_tlpA_ promoter will block the transcription of the *mCherry* reporter and thereby influence fluorescence. (B) Relative mCherry fluorescence values (mCherry fluorescence/OD_600_) of wild type and recombinant *L. rhamnosus* GR‐1 (pAMB6500, pAMB6505, pAMB6506) are shown. Significance testing was performed against the wild type strain, significance levels (*p‐value < 0.05, **p‐value < 0.005 and ***p‐value < 0.0005) and fluorescence fold changes are depicted on the graphs. (C) Optical density at 600 nm (OD_600_) and mCherry fluorescence of medium, *L. rhamnosus* GR‐1 wild type, *L. rhamnosus* GR‐1 pAMB6500, *L. rhamnosus* GR‐1 pAMB6505 and *L. rhamnosus* GR‐1 pAMB6506 were monitored over 24 h in a plate reader experiment. Mean values with standard deviations of three independent biological replicates are depicted in the graphs.

Apart from constitutive promoters, other genetic parts, such as transcriptional repressors, are key parts for tuning the genetic programmability of bacteria (Buson et al. 2024). Here, we assessed the performance of the Rep repressor from mv4 phage infecting *L. delbrueckii* , whose functionality was validated previously in *L. plantarum* (Blanch‐Asensio et al. 2024; Coddeville et al. 2007). In the relevant construct pAMB6506, the rep expression was controlled by the constitutive P_48_ promoter (Figure 2A), a promoter of moderate activity in *L. rhamnosus* GR‐1, and the P_tlpA_ promoter was flanked by upstream and downstream rep‐binding operator sites. To incorporate the repressor in the plasmid and obtain a mutation‐free plasmid, a time‐efficient direct cloning protocol was employed for *L. rhamnosus* GR‐1, inspired by a similar strategy previously implemented in *L. plantarum* (Blanch‐Asensio, Dey, and Sankaran 2023; Blanch‐Asensio et al. 2024).

The effectiveness of the Rep repressor in *L. rhamnosus* GR‐1 was evaluated by measuring the *mCherry* fluorescence levels using a plate reader (Figure 2B), and significance testing was performed against the wild type background. First, the effect of the operator sites flanking the P_tlpA_ promoter was assessed by measuring the mCherry fluorescence levels (pAMB6505). This resulted in a prominent 24.4‐fold decrease in the mCherry fluorescence, possibly as a consequence of altering the natural 5′ untranslated region (UTR) of the promoter (Figure 2B). Next, the effect of the repressor was evaluated by assessing mCherry fluorescence in the *L. rhamnosus* GR‐1 strain carrying the Rep‐encoding gene (pAMB6506). Minimal mCherry fluorescence was measured in the presence of the repressor, with a reduction of over 94.7% compared to the non‐rep encoding *L. rhamnosus* GR‐1 strain (pAMB6500). As a result, 5.3% leakiness of the repressor could be observed (Figure 2C).

Furthermore, we ran a kinetics study to explore both the effect of the rep repressor on bacterial growth and the stability of repression over time (Figure 2C). Rep exhibited minimal impact on growth, as the growth curves of the rep‐encoding strain (pAMB6506) were very similar to *L. rhamnosus* GR‐1 not encoding the repressor (pAMB6500) (Figure 2C). The mCherry fluorescence measurements showed that gene repression was remarkably robust over time, as comparable fluorescent levels to wild‐type bacteria were recorded during the course of 24 h.

Altogether, these results indicate that the Rep repressor proved efficient at repressing the P_tlpA_ promoter, thus leading to the identification of a functional transcriptional repressor that can be incorporated into the genetic toolbox of *L. rhamnosus* GR‐1.

### 3.3 Generation of Additional Fluorescent *L. rhamnosus* GR‐1 Strains Producing mScarlet3 and sfGFP

Expanding the library of functional fluorescent reporters in *L. rhamnosus* GR‐1 would facilitate the study of multiple engineered populations simultaneously. Therefore, the functionality of different fluorescent proteins in *L. rhamnosus* GR‐1 was explored under the control of the strong P_tlpA_ promoter, including the green fluorescent protein sfGFP and the red fluorescent protein mScarlet3 (Table 2). The functionality of these fluorescent proteins has not yet been tested in *L. rhamnosus* GR‐1.

**FIGURE 3:**
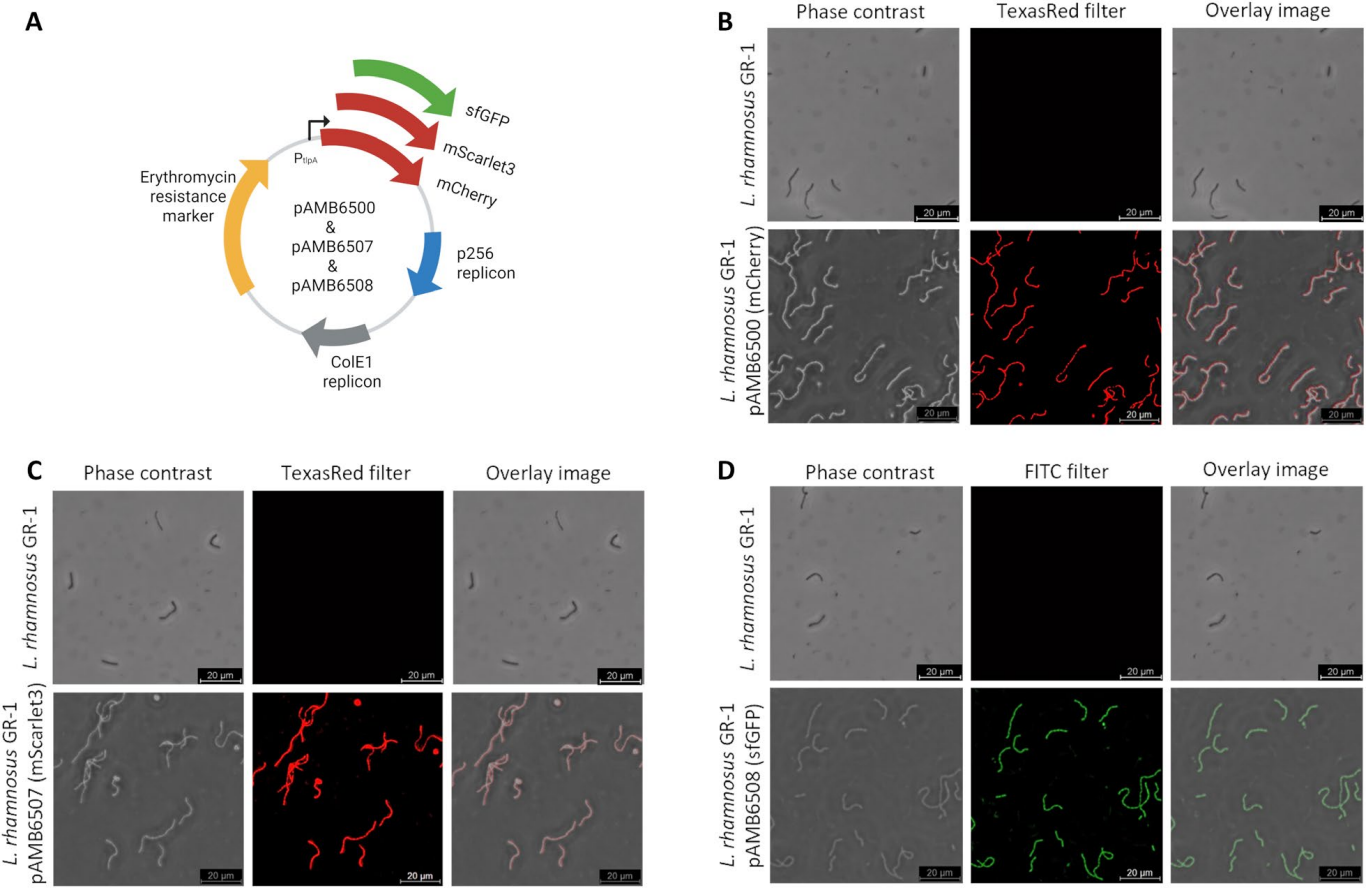
Fluorescence microscopy images of different red (mCherry, mScarlet3) and green (sfGFP) fluorescent reporters in *Lacticaseibacillus rhamnosus* GR‐1 under control of P_tlpA_. (A) Overview of plasmid design. Fluorescent reporters mCherry (pAMB6500), mScarlet3 (pAMB6507) and sfGFP (pAMB6508) were cloned downstream of P_tlpA_ in the p256 plasmid backbone. (B) Phase contrast, fluorescence image and overlay of *L. rhamnosus* GR‐1 pAMB6500 expressing mCherry fluorescent protein compared to the wild type bacteria. (C) Phase contrast, fluorescence image and overlay of *L. rhamnosus* GR‐1 pAMB6507 expressing mScarlet3 fluorescent protein compared to wild type bacteria. (D) Phase contrast, fluorescence image and overlay of *L. rhamnosus* GR‐1 pAMB6508 expressing sfGFP fluorescent protein compared to wild type bacteria. For mCherry and mScarlet3 visualization, the TexasRed filter was used. For sfGFP visualization, the FITC filter was used. 40× magnification was used for visualization of the bacteria.

The genes for the mScarlet3 and sfGFP fluorescent reporter proteins were cloned downstream of the constitutive P_tlpA_ promoter in the p256 screening vector (Figure 3A), resulting in plasmids pAMB6507 (mScarlet3) and pAMB6508 (sfGFP). Fluorescence microscopy revealed clear functionality of mCherry (Figure 3B), mScarlet3 (Figure 3C) and sfGFP (Figure 3D) in *L. rhamnosus* GR‐1, while no background signal could be detected from non‐engineered wild type *L. rhamnosus* GR‐1.

### 3.4 Fluorescent *L. rhamnosus* GR‐1 for Investigating Microbe‐Microbe Interactions and Bacterial Population Dynamics

**FIGURE 4:**
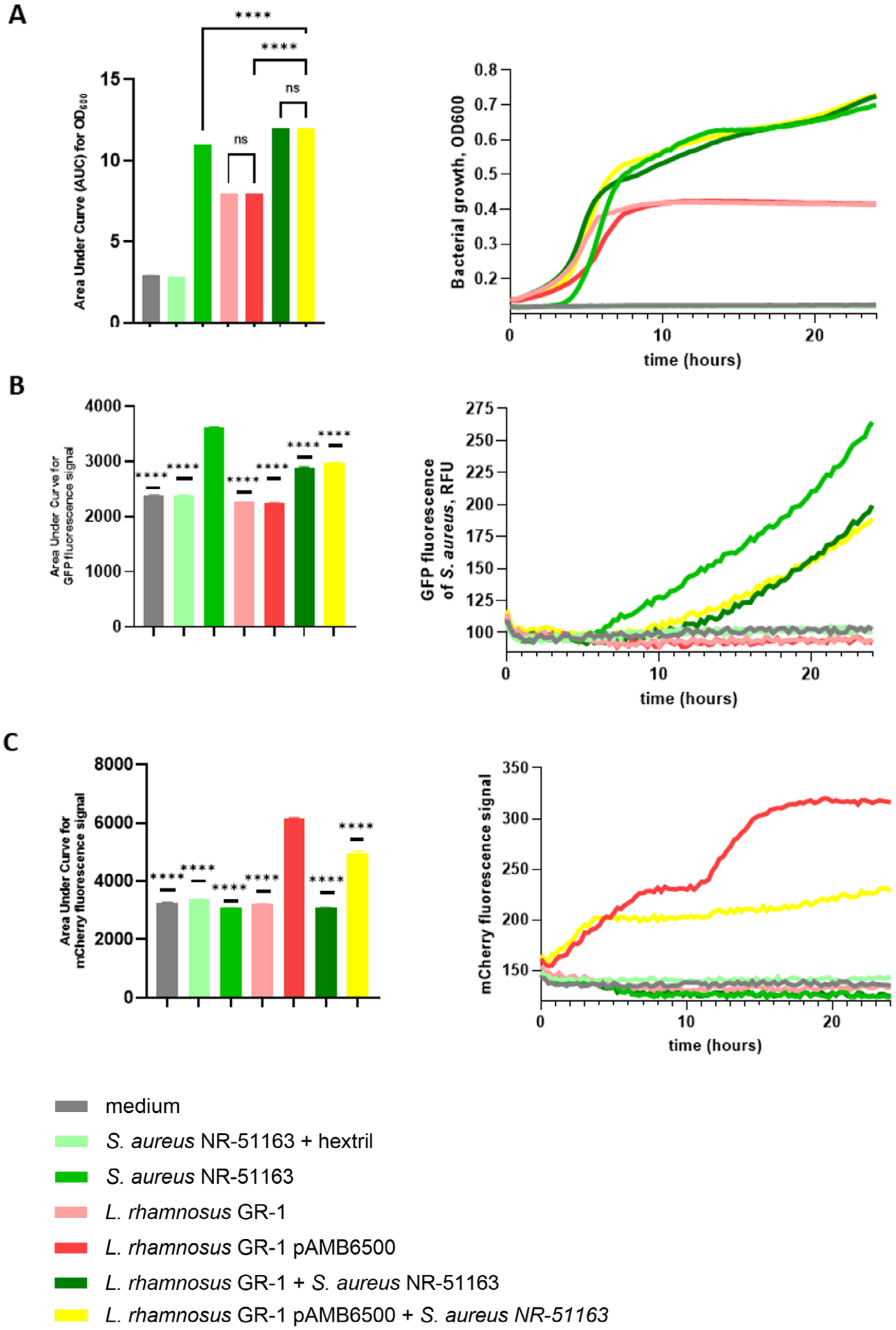
Multi‐strain time‐course growth competition experiment of *Lacticaseibacillus rhamnosus* GR‐1 (pAMB6500) with pathogenic *Staphylococcus aureus* NR‐51163. (A) Overall bacterial growth (OD_600_) was monitored over time. (B) GFP fluorescence was used to track the growth of *S. aureus* NR‐51163. Ex (nm): 485/20 and Em (nm): 528/20 filters were used in the plate reader. (C) mCherry fluorescence was used to track the growth of *L. rhamnosus* GR‐1 pAMB6500. Ex (nm): 580/50 and Em (nm): 635/32 filters were used in the plate reader. Area under curve (AUC) data and time‐course measurements are depicted. Measurements were taken for 24 h every 15 min, and the experiment was performed in biological triplicate. Hextril, an antiseptic agent, was used as a positive control to inhibit *S. aureus* NR‐51163. Medium was included as a negative control. Mean values with standard deviations (for AUC) are depicted in the graph. Significance testing was performed against *S. aureus* NR‐51163 for GFP fluorescence measurements and against *L. rhamnosus* GR‐1 pAMB6500 for mCherry fluorescence measurements. Significance levels are indicated on the graphs: ****p‐value < 0.00005 and ns, not significant.

To illustrate the applicability of fluorescent *Lactobacillaceae* in functional microbiome research, including studying bacterial community dynamics, a multi‐strain time‐course growth inhibition experiment between *L. rhamnosus* GR‐1 strains (wild type and pAMB6500) and pathogenic GFP‐expressing *Staphylococcus aureus* NR‐51163 was performed for 24 h. OD_600_ was measured to follow the overall bacterial growth (Figure 4A), combined with GFP fluorescence to follow growth of *S. aureus* NR‐51163 producing GFP (Figure 4B) and mCherry fluorescence to monitor growth kinetics of *L. rhamnosus* GR‐1 pAMB6500 (Figure 4C). Area Under the Curve (AUC) was calculated as a summary growth metric for each condition (Duan et al. 2012) and shown together with time‐course measurements over 24 h (Figure 4).

For OD_600_ bacterial growth measurements (Figure 4A), no significant difference was present between wild type *L. rhamnosus* GR‐1 and *L. rhamnosus* GR‐1 pAMB6500, indicating minimal metabolic burden of plasmid maintenance on *L. rhamnnosus* GR‐1. Similarly, there was no significant growth difference between wild type *L. rhamnosus* GR‐1 co‐cultured with *S. aureus* NR‐51163 and *L. rhamnosus* GR‐1 pAMB6500 co‐cultured with *S. aureus* NR‐51163. In contrast, higher bacterial growth was observed in combination cultures compared to single cultures (*p* < 0.00005). For GFP fluorescence measurements (Figure 4B), significant reduction was observed in combination cultures compared to *S. aureus* NR‐51163 in single culture, presumably due to competitive effects between (recombinant) *L. rhamnosus* GR‐1 (pAMB6500) and *S. aureus* NR‐51163. This inhibitory effect on the growth of *S. aureus* NR‐51163 by *L. rhamnosus* GR‐1 and *L. rhamnosus* GR‐1 pAMB6500 was also confirmed by CFU counting of single and combination cultures after this 24 h run (Figure S4). *S. aureus* NR‐51163 reached 2.47 ± 0.50 × 10^9^ CFU/mL in single culture after 24 h compared to 4.67 ± 2.76 × 10^7^ CFU/mL and 4.57 ± 1.62 × 10^7^ CFU/mL when grown in combination with *L. rhamnosus* GR‐1 and *L. rhamnosus* GR‐1 pAMB6500, respectively. As a result, there is a significant decrease in growth of *S. aureus* NR‐51163 of 2.47 ± 0.50 × 10^9^ CFU/mL by *L. rhamnosus* GR‐1. Similarly, *L. rhamnosus* GR‐1 pAMB6500 reduced the growth of *S. aureus* NR‐51163 with 2.41 ± 0.50 × 10^9^ CFU/mL compared with *S. aureus* NR‐51163 grown in single culture. In addition, a bacterial spot assay was performed as a conventional method to confirm the inhibitory activity of *L. rhamnosus* on *S. aureus* growth (Figure S5). Inhibition zones of 22.88 ± 0.88 nm and 23.14 ± 2.45 nm were measured for *L. rhamnosus* GR‐1 and *L rhamnosus* GR‐1 pAMB6500 which are comparable with reported halos for reference strain *L. rhamnosus* GG confirming the antimicrobial activity of *L. rhamnosus* against *S. aureus*. High mCherry values were present for *L. rhamnosus* GR‐1 pAMB6500 as a single culture or in co‐culture with *S. aureus* NR‐51163, with the signal being higher for the single culture condition (*p* < 0.00005) (Figure 4C). This proof‐of‐concept experiment illustrates the applicability of fluorescent reporters to track individual strains in mixed cultures.

## 4 Discussion

Expanding the genetic toolbox of probiotic *Lactobacillaceae* facilitates studying their probiotic properties and mechanisms of action (Douillard and de Vos 2014; Van Pijkeren and Britton 2012) as well as elevating their potential in synthetic biology (Bravo and Landete 2017; Goh and Barrangou 2019). The range of genetic tools for *L. rhamnosus* has remained limited to date (Petrova et al. 2016, 2018) compared to other well‐studied *Lactobacillaceae* strains such as *L. plantarum* WCFS1, or model strains such as *E. coli* . In this study, we expanded the genetic toolbox of *L. rhamnosus* GR‐1 and *L. rhamnosus* GG by demonstrating the activity of several constitutive promoters. By combining these findings with fluorescence reporter analysis and an intermediate‐free direct cloning protocol, we engineered *L. rhamnosus* GR‐1 strains with visible constitutive red and green fluorescence and showed their functional relevance for microbiome research by a proof‐of‐concept time course experiment with pathogenic *S. aureus* NR‐51163.

A key achievement was the successful implementation of the strong *Salmonella*‐derived P_tlpA_ promoter, which showed significant, although strain‐dependent, activity in *L. rhamnosus* GR‐1 and *L. rhamnosus* GG. Interestingly, P_tlpA_ exhibited ~20‐fold higher promoter activity in *L. rhamnosus* GR‐1 compared to the previously established nisin‐inducible promoter P_nisA_ in the NICE system that served as reference for comparison in our study (Spacova et al. 2018). The visually pink colonies demonstrate that P_tlpA_ acts as a strong constitutive promoter in *L. rhamnosus* GR‐1. Another constitutive promoter, P_48_, showed moderate activity in both *L. rhamnosus* strains. P_tec_, P_cpg_ and P_23_ did not yield an observable fluorescence signal for both *L. rhamnosus* strains despite their performance in other *Lactobacillaceae* such as *L. plantarum* (Blanch‐Asensio et al. 2024; Dey et al. 2023). P_tlpA_ also showed differences in promoter activity between the closely related *L. rhamnosus* GR‐1 and *L. rhamnosus* GG, showing considerably higher activity (8.6 fold) in *L. rhamnosus* GR‐1, which can possibly be explained by a different cellular environment (McCracken et al. 2000; Tauer et al. 2014).

To confirm the possibility of using *L. rhamnosus* GR‐1 pAMB6500 in long‐term applications, we demonstrated the stability of P_tlpA_‐driven mCherry expression over 3 days of subculturing in both selective and non‐selective conditions. In addition, plasmid retention was assessed based on antibiotic resistance and demonstrated stable retention over 3 days. However, the lack of single‐cell resolution limits conclusions on population heterogeneity, such as variation in plasmid copy number between cells, which could be addressed using flow cytometry (Shao et al. 2021). Moreover, temperature‐dependent activity of P_tlpA_ in *L. rhamnosus* GR‐1 was observed. A similar trend was described for *L. plantarum* WCFS1 with a 5‐fold increase in expression from 31°C to 39°C (Dey et al. 2023). This high promoter activity of P_tlpA_ at 37°C in *L. rhamnosus* GR‐1 makes this promoter suitable for both industrial and therapeutic applications, particularly for conditions involving physiological temperatures.

To strategically modulate the constitutive expression levels of P_tlpA_ in *L. rhamnosus* GR‐1, we evaluated the functionality of a phage‐derived repressor, derived from the mv4 bacteriophage infecting *L. delbrueckii*, responsible for controlling lytic and lysogenic life cycles of the phage (Blanch‐Asensio et al. 2024). To our knowledge, this is the first study reporting a functional repressor for *L. rhamnosus* GR‐1. Blanch‐Asensio et al. (2024) performed a rigorous screening for repressors of *Lactobacillaceae‐*infecting phages, and reported the functioning of this repressor in *L. plantarum* WCFS1 with repression capacity of > 95%. Here, similar repression levels (94.7%) were observed in *L. rhamnosus* GR‐1, showing promising potential to be included in future work focusing on gene expression regulation in *L. rhamnosus* . For example, the Rep repressor can be incorporated in gene circuits based on inducible systems functional in lactobacilli and therefore invert their outputs (Blanch‐Asensio et al. 2025; De Baets et al. 2024; Heiss et al. 2016). Moreover, this repressor can be further engineered to sense and respond to external stimuli such as light, temperature or pH and thus develop novel inducible systems in *L. rhamnosus* (Dimas et al. 2019). However, reduced (24.4 fold) promoter activity was observed upon addition of the operator sequences suggesting interference with RNA polymerase recognition or disruption of promoter architecture. Further work could include optimizing operator positioning and spacing in order to increase basal expression levels (Garcia et al. 2012). These approaches are particularly relevant in synthetic biology, where fine‐tuning of gene expression is essential to balance protein production with cellular fitness, especially in industrial fermentation or therapeutic context.

As a next step, we developed constitutively red and green fluorescent *L. rhamnosus* GR‐1 based on the strong P_tlpA_ promoter evaluated by fluorescence microscopy. The availability of spectrally distinct reporters facilitates genetic tool evaluation using fluorescence as readout and supports research focusing on host‐microbe or microbe‐microbe interactions, adhesion and colonization patterns (Dean and Palmer 2014; Landete et al. 2016). Visualization of fluorescent *Lactobacillaceae* with fluorescence microscopy is highly relevant for functional microbiome research but requires higher per‐cell fluorescence than what is needed for the detection of bulk fluorescence in plate reader assays. Due to the strong P_tlpA_ promoter activity, fluorescent *L. rhamnosus* GR‐1 producing mCherry, mScarlet3 and sfGFP could be visualized with fluorescence microscopy, which has not been reported yet for constitutively fluorescent *L. rhamnosus* GR‐1 to the best of our knowledge. Up to now, only fluorescent *L. rhamnosus* GR‐1 has been successfully constructed using the inducible NICE system, which hampers their application for longitudinal in vitro studies and in vivo monitoring (Spacova et al. 2018) due to the requirement for nisin induction and unpredictability in induction pattern caused by heterogeneity in uptake and delivery of nisin (Geoffroy et al. 2000). In other studies, fluorescent reporter expression under the control of constitutive promoters in *L. rhamnosus* did not yet yield detectable fluorescence signals. For example, no GFP expression based on P_ldhL_ originating from *L. plantarum* WCFS1 could be detected in *L. rhamnosus* GG (De Keersmaecker et al. 2006); and for both *L. rhamnosus* GG and GR‐1, endogenous promoters (P_msp1_, P_dlt_ and P_spaCBA_) did not show detectable fluorescence for mTagBFP2 and mCherry (Spacova et al. 2018). By using a strong promoter, we obtained higher expression levels compared to previously used endogenous and *Lactobacillaceae‐*derived promoters allowing the construction of fluorescent *L. rhamnosus* GR‐1 expressing mCherry, mScarlet3 and sfGFP. The sfGFP reporter has been used for promoter evaluation in *L. paracasei* and *L. plantarum* , but to date, its functionality in *L. rhamnosus* has not been shown yet (Fisher and DeLisa 2008; Pédelacq et al. 2006). Notably, differences in fluorescent signal may reflect the pH sensitivity of these fluorescent proteins. As the culture medium was not buffered, *L. rhamnosus* GR‐1 overnight cultures reached a low pH (~3.5–4) due to lactic acid production, which likely affected sfGFP stability (Fisher and DeLisa 2008). In contrast, mCherry and mScarlet3 retain fluorescence at a broader pH range making them potentially more suitable for applications with *L. rhamnosus* or other lactic acid producing bacteria (Gadella et al. 2023; Shaner et al. 2004; Tadimarri et al. 2026).

The broad relevance of fluorescent *L. rhamnosus* GR‐1 was illustrated by a proof‐of‐concept time‐course inhibition experiment between pathogenic *S. aureus* NR‐51163 producing GFP and *L. rhamnosus* GR‐1 pAMB6500 producing mCherry under P_tlpA_. A key limitation of time‐course inhibition analysis of pathogens by *Lactobacillaceae* is that absorbance measurements cannot distinguish between the pathogen and the *Lactobacillaceae* strain. As a result, these assays are typically performed with the cell‐free culture supernatant of the beneficial strain instead of live bacteria (Goyal and Kannan 2018; Scillato et al. 2021). Co‐culturing of fluorescent bacteria allows tracking of individual populations in mixed culture, which can be broadly applied in functional microbiome research, unravelling community dynamics (Aherne et al. 2024). In addition, fluorescence production offers a more appealing strategy in research to monitor the bacterial dynamics instead of a time‐consuming quantitative PCR (Spacova, De Boeck, et al. 2023). Our experiment illustrated the efficient use of fluorescence to track separately the growth of *L. rhamnosus* GR‐1 pAMB6500 and *S. aureus* NR‐51163 by measuring mCherry and GFP fluorescence, respectively, over time. OD_600_ measurements were taken to track the overall growth of the mixed culture. For example, high OD_600_ values were reached for co‐culture *L. rhamnosus* GR‐1 pAMB6500 and *S. aureus* NR‐51163, but no insights into competitive or inhibitory effects can be derived from this data, illustrating the relevance of including fluorescence measurements. However, a two‐step increase of mCherry fluorescence could be observed for *L. rhamnosus* GR‐1 pAMB6500, which was not observed in the OD_600_ measurements. This can be explained by the maturation kinetics of mCherry, as fluorescence depends on a two‐step process involving protein folding and subsequent chromophore formation (Hebisch et al. 2013). For GFP fluorescence, a significant decrease in signal was observed in combination cultures with *L. rhamnosus* GR‐1 (wild type and pAMB6500) indicating the inhibitory effect of *L. rhamnosus* GR‐1 on the growth of *S. aureus* , which was independently confirmed by CFU/mL counts and a spot assay, yielding results in line with earlier studies (El‐Chami et al. 2022; Rodriguez et al. 2025; Spacova, O'Neill, and Lebeer 2020). Therefore, the combination of absorbance and fluorescence measurements is recommended to track bacterial growth dynamics. These constitutively fluorescent *L. rhamnosus* GR‐1 and the proof‐of‐concept experiment pave the way for further functional in vitro and in vivo microbiome research, including studying adhesion and colonization properties, as well as competitive or supporting community dynamics (Berlec et al. 2015; Stojanov et al. 2024; van Zyl et al. 2015).

For evaluation of the different genetic tools in this study, the replicative p256 backbone with an erythromycin resistance marker was used, limiting direct applicability of this plasmid in clinical settings. Future work could focus on genomic integration thereby ensuring robust and antibiotic‐free retention of genetic constructs (e.g., for expressing relevant industrial or therapeutic molecules). In addition, the development of antibiotic‐free selection strategies, such as auxotrophy (Bron et al. 2002), toxin‐antitoxin systems (Dey et al. 2023; Fedorec et al. 2019) or overlapping gene design (Leonard et al. 2026), can be used to eliminate antibiotic selection mechanisms. Such developments will be essential to enable translational use of *L. rhamnosus* expressing relevant molecules. These strategies are particularly relevant in the light of regulatory constraints, which increasingly restrict the use of antibiotic resistance markers in food‐grade and therapeutic microorganisms (Spacova, Binda, et al. 2023). Together, this future work would strengthen the translational potential of these genetic tools and support the implementation of *L. rhamnosus* as chassis in synthetic biology.

## 5 Conclusions

Overall, our results highlight the potential of *L. rhamnosus* GR‐1 and *L. rhamnosus* GG for both fundamental microbiome research and applied synthetic biology applications. Different tools already validated in well‐studied *L. plantarum* WCFS1 were now evaluated in *L. rhamnosus* GR‐1 and *L. rhamnosus* GG, both strains with industrial, medical and therapeutic potential across different body niches. Due to strain‐specific functionality, this study illustrates the challenge of creating a universal genetic engineering toolbox for *Lactobacillaceae* and emphasizes the need for tailoring genetic tools to a specific strain. Nevertheless, several constitutive promoters of varying strengths were obtained for *L. rhamnosus* GR‐1 and *L. rhamnosus* GG including the strong P_tlpA_ promoter. Using this promoter, we obtained a functional repressor system and generated visibly fluorescent red (mCherry and mScarlet3) and green (sfGFP) *L. rhamnosus* GR‐1. These genetic tools enable precise and effective engineering of this non‐model organism, thereby enhancing its industrial potential and facilitating functional microbiome research.

## Author Contributions

**Ilke Van Tente:** conceptualization, investigation, writing – original draft, methodology, visualization, writing – review and editing, formal analysis. **Sarah Lebeer:** writing – review and editing, funding acquisition, methodology, supervision, resources, conceptualization. **Irina Spacova:** supervision, conceptualization, writing – original draft, writing – review and editing, methodology, funding acquisition, resources. **Dieter Vandenheuvel:** writing – review and editing, methodology. **Tom Eilers:** supervision, writing – review and editing, conceptualization, methodology. **Marc Blanch‐Asensio:** conceptualization, investigation, writing – original draft, methodology, formal analysis, writing – review and editing. **Shrikrishnan Sankaran:** funding acquisition, writing – review and editing, conceptualization, resources, supervision, methodology.

## Funding

This study was supported by Fonds Wetenschappelijk Onderzoek (FWO SBO DeVeniR: S006424N), European Research Council (852600 awarded to SL), Interuniversitair Bijzonder Onderzoeksfonds ‐ Inter‐Universitary Special Research Fund of Flanders (iBOF) (iBOF/21/092 POSSIBL project), Deutsche Forschungsgemeinschaft (455063657), Leibniz Science Campus Living Therapeutic Materials (LifeMat) and Universiteit Antwerpen.

## Conflicts of Interest

S.L. declares to be a voluntary academic board member of the International Scientific Association on Probiotics and Prebiotics (ISAPP, http://www.isappscience.org), cofounder of YUN and scientific advisor for Freya Biosciences. She declares research funding from YUN, Bioorg, Puratos, DSM I‐Health and Lesaffre/Gnosis. T.E. is partially funded through an industrial research VLAIO grant not related to this work. D.V. is cofounder of the Belgian Society for Viruses of Microbes (BSVoM, https://www.bsvom.be). None of these organizations or companies were involved in designing of the experiments and data analysis. The other authors declare no conflicts of interest.

## Supporting information


**Table S1:** Bacterial strains and plasmids used in this study. For each bacterial strain and plasmid, a description and references/sources are included. CmR, chloramphenicol resistance; EryR, erythromycin resistance.
**Table S2:** Primers used in this study. Sequence and description are added for each primer.
**Table S3:** Fold changes in promoter activity compared to wild type in *Lacticaseibacillus rhamnosus* GR‐1 and adjusted *p*‐values.
**Table S4:** Fold changes in promoter activity compared to wild type in *Lacticaseibacillus rhamnosus* GG and adjusted *p*‐values.
**Figure S1:** Comparison of promoter activity of P_tlpA_, P_48_ and P_nisA_ between *Lacticaseibacillus rhamnosus* GR‐1 and *L. rhamnosus* GG. Different constitutive promoters that showed significant *mCherry* expression (P_tlpA_, P_48_ and P_nisA_) in *L. rhamnosus* GR‐1 and *L. rhamnosus* GG are shown. P_tlpA_ and P_48_ were cloned upstream of the *mCherry* gene in the screening p256 plasmid backbone. P_nisA_ was present upstream of *mCherry* in a derivative of pMEC45. Fluorescence values were corrected for bacterial growth, measured as OD_600_ and shown on the figure as ‘Relative mCherry fluorescence units’. Mean values with standard deviations of three independent biological replicates are depicted in the graph. Significance testing was performed using multiple unpaired *t*‐tests with Holm‐Šídák method, and significance levels are indicated on the graphs: *p < 0.05 and ns: not significant. Fold changes of promoter activity between *L. rhamnosus* GR‐1 and *L. rhamnosus* GG are also depicted on the graph.
**Figure S2:** Assessing plasmid retention based on antibiotic resistance of pAMB6500 in *Lacticaseibacillus rhamnosus* GR‐1. Overnight cultures of *L. rhamnosus* GR‐1 containing pAMB6500 were sub cultured every 24 h in media without antibiotics. Cultures were plated in serial dilutions on plates containing antibiotics and plates without antibiotics. Colony Forming Units (CFUs) were counted and ratio of CFUs in selective conditions against CFUs in non‐selective conditions was plotted in percentages on the *y*‐axis. This experiment was conducted on three consecutive days. Mean values with standard deviations of three independent biological replicates are depicted in the graph. Significance testing was performed using one‐way ANOVA with Tukey correction but no significant (ns) differences between conditions could be detected.
**Figure S3:** Native thermo‐responsiveness of P_tlpA_ in *Lacticaseibacillus rhamnosus* GR‐1. Recombinant *L. rhamnosus* GR‐1 containing the pAMB6500 plasmid was grown at different temperatures (30°C, 33°C, 37°C and 39°C), after which mCherry fluorescence was measured (A). Fluorescence values were corrected for bacterial growth, measured as OD_600_ and shown on the figure as ‘Relative mCherry fluorescence units’. Mean values with standard deviations of three independent biological replicates are depicted in the graph. Significance testing was performed against *L. rhamnosus* GR‐1 pAMB6500 grown at 37°C using Welch's ANOVA with Dunnett T3 correction. Significance levels (*p‐value < 0.05, **p‐value < 0.005) are indicated on the graph. No significant differences in OD_600_ of overnight cultures grown at different temperatures could be observed (B).
**Figure S4:** Colony Forming Units (CFU) comparison of *Staphylococcus aureus* NR‐511603, *Lacticaseibacillus rhamnosus* GR‐1 and *L. rhamnosus* GR‐1 pAMB6500 grown in single and combination cultures. (A) Comparison of bacterial growth of *S. aureus* NR‐511603 grown as single culture compared to when grown combined with *L. rhamnosus* GR‐1 or *L. rhamnosus* GR‐1 pAMB6500. Significant growth reduction can be observed for *S. aureus* NR‐51163 when combined with *L. rhamnosus* GR‐1 or *L. rhamnosus* GR‐1 pAMB6500. (B) Comparison of *L. rhamnosus* GR‐1 and *L. rhamnosus* GR‐1 pAMB6500 grown as a single culture compared to when grown in combination with *S. aureus* NR‐51163. No significant growth difference could be observed between single and combination culture conditions. Combination cultures were spotted in serial dilution on selective agar containing BHI with 10 μg/mL trimethoprim for *S. aureus* NR‐51163, MRS with 10 μg/mL erythromycin for *L. rhamnosus* GR‐1 pAMB6500. For wild type *L. rhamnosus* GR‐1, MRS agar plates without antibiotics were used. Mean values with standard deviations of three independent biological replicates are depicted in the graph. Significance testing was performed using Welch's ANOVA with Dunnett T3 correction and significance levels are indicated on the graphs: *p < 0.05 and ns, not significant.
**Figure S5:** Bacterial spot assays investigating inhibitory effect of *Lacticaseibacillus rhamnosus* GR‐1 and *L. rhamnosus* GR‐1 pAMB6500 on the growth of *Staphylococcus aureus* NR‐51163. Spots of overnight cultures of *L. rhamnosus* GR‐1 (wild type and pAMB6500) were added to the plate and incubated at 37°C for 48 h. After that, soft agar top layer containing *S. aureus* NR‐51163 was added to the plate. After 24 h incubation, the plate was visualized showing clear halos indicating inhibition of *S. aureus* growth by *L. rhamnosus* strains (A). Hextril and clindamycin were included as positive controls. (B) Diameter of inhibition zones (halos) in nanometre (nm) were measured and plotted. Three independent biological replicates were included.

## Data Availability

The data that support the findings of this study are available from the corresponding author upon reasonable request.
