# Supplementary material for: Genetic Toolbox Expansion Enables Constitutively Fluorescent Lacticaseibacillus rhamnosus for Functional Microbiome Research
Source: Microb Biotechnol. 2026 Jul 9;19(7):e70405. doi: 10.1111/1751-7915.70405 (PMC13350346; doi:10.1111/1751-7915.70405)
Supplement: Supplementary file 1 — Table S1: Bacterial strains and plasmids used in this study. For each bacterial strain and plasmid, a description and references/sources are included. CmR, chloramphenicol resistance; EryR, erythromycin resistance. Table S2: Primers used in this study. Sequence and description are added for each primer. Table S3: Fold changes in promoter activity compared to wild type in Lacticaseibacillus rhamnosus GR‐1 and adjusted p‐values. Table S4: Fold changes in promoter activity compared to wild type in Lacticaseibacillus rhamnosus GG and adjusted p‐values. Figure S1: Comparison of promoter activity of PtlpA, P48 and PnisA between Lacticaseibacillus rhamnosus GR‐1 and L. rhamnosus GG. Different constitutive promoters that showed significant mCherry expression (PtlpA, P48 and PnisA) in L. rhamnosus GR‐1 and L. rhamnosus GG are shown. PtlpA and P48 were cloned upstream of the mCherry gene in the screening p256 plasmid backbone. PnisA was present upstream of mCherry in a derivative of pMEC45. Fluorescence values were corrected for bacterial growth, measured as OD600 and shown on the figure as ‘Relative mCherry fluorescence units’. Mean values with standard deviations of three independent biological replicates are depicted in the graph. Significance testing was performed using multiple unpaired t‐tests with Holm‐Šídák method, and significance levels are indicated on the graphs: *p < 0.05 and ns: not significant. Fold changes of promoter activity between L. rhamnosus GR‐1 and L. rhamnosus GG are also depicted on the graph. Figure S2: Assessing plasmid retention based on antibiotic resistance of pAMB6500 in Lacticaseibacillus rhamnosus GR‐1. Overnight cultures of L. rhamnosus GR‐1 containing pAMB6500 were sub cultured every 24 h in media without antibiotics. Cultures were plated in serial dilutions on plates containing antibiotics and plates without antibiotics. Colony Forming Units (CFUs) were counted and ratio of CFUs in selective conditions against CFUs in non‐selective conditi [file MBT2-19-e70405-s001.docx]

**Supplementary Material**

Genetic Toolbox Expansion Enables Constitutively Fluorescent *Lacticaseibacillus rhamnosus* for Functional Microbiome Research

**Ilke Van Tente^1^,** Marc Blanch Asensio^2^, Tom Eilers^1^, Dieter Vandenheuvel^1^, Sarah Lebeer^1,3^, **Shrikrishnan Sankaran** ^†^**^2^ & Irina Spacova**^†^**^1*^**

^1^ Laboratory of Applied Microbiology and Biotechnology, Department of Bioscience Engineering, University of Antwerp, Campus Groenenborger, 2020 Antwerp, Belgium
^2^ Bioprogrammable Materials, INM-Leibniz Institute for New Materials, Campus D2 2, 66123 Saarbrücken, Germany
^3^ U-MaMi Centre of Excellence, University of Antwerp, 2020 Antwerp, Belgium

† These authors contributed equally to the work and share senior authorship

**Table S1.** **Bacterial strains and plasmids used in this study.** For each bacterial strain and plasmid, a description and references/sources are included. EryR: erythromycin resistance; CmR: chloramphenicol resistance.

| Bacterial strains | Description | References and/or sources |
| --- | --- | --- |
| *Lacticaseibacillus rhamnosus* GR-1 (ATCC 5582) | Wild-type human urogenital isolate | (Reid et al., 2001) |
| *Lacticaseibacillus rhamnosus* GG (ATCC 53103) | Wild-type human gastrointestinal isolate | (Kankainen et al., 2009) |
| CMPG11262 | FAJ1905 strain (chromosomal insertion of pMEC10 in *attB* (phage mv4)) of *L. rhamnosus* GG carrying pCMPG11261 with *mCherry;* EryR and CmR | (Spacova et al., 2018) |
| CMPG11265 | CMPG11259 strain (chromosomal insertion of pMEC10 in *attB* (phage mv4)) strain of *L. rhamnosus* GR-1 carrying pCMPG11261 with *mCherry*; EryR and CmR | (Spacova et al., 2018) |
| *Escherichia coli* DH5α | Commonly used *E. coli* laboratory strain | New England Biolabs |
| *Staphylococcus aureus* NR-51163 | *S. aureus* containing the green fluorescent protein (GFP) reporter plasmid pSGFPS1, a derivative of *E. coli* staphylococcal shuttle vector pKK30 | BEI Resources, NIAID, NIH  (Catalog No. NR-51163) |
| Plasmids | **Description** | **References and/or sources** |
| pAMB6500 | Derivative of p256 containing *mCherry* cloned downstream of P_tlpA_; EryR | (Dey et al., 2023) |
| pAMB6501 | Derivative of p256 containing *mCherry* cloned downstream of P_tec_; EryR | (Blanch-Asensio et al., 2024) |
| pAMB6502 | Derivative of p256 containing *mCherry* cloned downstream of P_cpg_; EryR | (Blanch-Asensio et al., 2024) |
| pAMB6503 | Derivative of p256 containing *mCherry* cloned downstream of P_48_ ; EryR | (Dey et al., 2023) |
| pAMB6504 | Derivative of p256 containing *mCherry* cloned downstream of P_23_ ; EryR | (Dey et al., 2023) |
| pAMB6505 | Derivative of p256 containing *mCherry* cloned downstream of P_tlpA_ with operator sites; EryR | (Blanch-Asensio et al., 2024) |
| pAMB6506 | Derivative of p256 containing Rep repressor downstream of P_48_ promoter and *mCherry* cloned downstream of P_tlpA_; EryR | (Blanch-Asensio et al., 2024) |
| pAMB6507 | Derivative of p256 containing *mScarlet3* cloned downstream of P_tlpA_; EryR | This study |
| pAMB6508 | Derivative of p256 containing *sfGfp* cloned downstream of P_tlpA_; EryR | This study |
| pCMPG11262 | Derivative of pMEC45 containing *mCherry* cloned from pRSETb-*mCherry* downstream *nisA* promoter; CmR | (Spacova et al., 2018) |

**Table S2.** **Primers used in this study.** Sequence and description are added for each primer.

| Primer | Sequence | Description |
| --- | --- | --- |
| gSL426 | \| GAAGGAGATATACCATGATGATGGACTCAACAGAAGCC \| \| --- \| | Forward primer insert (mScarlet3) pAM6507 |
| gSL427 | \| CCAAGGGGTTATGCTAGTTATT \| \| --- \| | Reverse primer insert (mScarlet3) pAMB6507 |
| gSL428 | AATAACTAGCATAACCCC | Forward primer vector pAMB6507 |
| gSL429 | CATCATGGTATATCTCCT | Reverse primer vector pAMB6507 |
| gSL430 | CTAAAGGGAATGGAGACC | Forward primer insert (sfGFP) pAMB6508 |
| gSL431 | \| AGTCTCGGACATTCTGCT \| \| --- \| | Reverse primer insert (sfGFP) pAMB6508 |
| gSL432 | \| GGAGCAGAATGTCCGAGAC \| \| --- \| | Forward primer vector pAMB6508 |
| gSL433 | \| CCGGTCTCCATTCCCTTTAG \| \| --- \| | Reverse primer vector pAMB6508 |
| gSL434 | \| CCGTTAGCGTAGTAGTAGC \| \| --- \| | Forward primer for linearisation pAMB6505 and pAMB6506 |
| gSL435 | CTTCAAAGGGTCAACAGC | Reverse primer for linearisation pAMB6505 and pAMB6506 |
| gSL436 | CGTTACTAAAGGGAATGGAG | Forward confirmation primer flanking multiple cloning site of constructed plasmids |
| gSL437 | CAGTGGAACGAAAACTCACG | Reverse confirmation primer flanking multiple cloning site of constructed plasmids |

**Table S3. Fold changes in promoter activity compared to wild type in *L. rhamnosus* GR-1 and adjusted p-values.**

| Promoter | Fold change compared to wild type | (Adjusted) p-value |
| --- | --- | --- |
| P_tlpA_ | 79.67 ± 2.47 | < 0.0001 |
| P_tec_ | 2.34 ± 0.33 | > 0.9999 |
| P_cpg_ | 1.34 ± 0.02 | 0.9950 |
| P_48_ | 2.95 ± 0.25 | 0.0001 |
| P_23_ | 1.65 ± 0.03 | 0.7964 |
| P_nisA_ | 3.96 ± 0.12 | 0.0002 |

**Table S4. Fold changes in promoter activity compared to wild type in *L. rhamnosus* GG and adjusted p-values.**

| Promoter | Fold change compared to wild type | (Adjusted) p-value |
| --- | --- | --- |
| P_tlpA_ | 8.24 ± 1.25 | 0.0023 |
| P_tec_ | 1.05 ± 0.05 | 0.4539 |
| P_cpg_ | 1.17 ± 0.01 | > 0.9999 |
| P_48_ | 3.46 ± 0.33 | 0.1071 |
| P_23_ | 1.42 ± 0.11 | > 0.9999 |
| P_nisA_ | 3.44 ± 0.09 | 0.0184 |


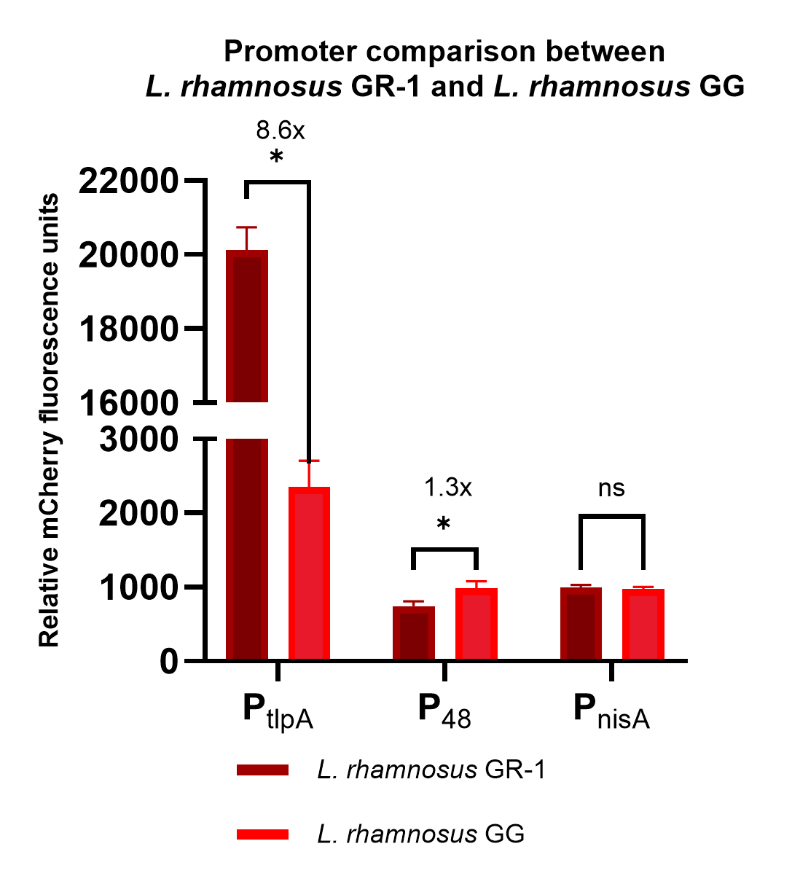


**Figure S1. Comparison of promoter activity of P_tlpA_, P_48_ and P_nisA_ between *L. rhamnosus* GR-1 and *L. rhamnosus* GG.** Different constitutive promoters that showed significant *mCherry* expression (P_tlpA_, P_48_ and P_nisA_) in *L. rhamnosus* GR-1 and *L. rhamnosus* GG are shown. P_tlpA_ and P_48_ were cloned upstream of the *mCherry* gene in the screening p256 plasmid backbone. P_nisA_ was present upstream of *mCherry* in a derivative of pMEC45. Fluorescence values were corrected for bacterial growth, measured as OD_600_ and shown on the figure as ‘Relative mCherry fluorescence units’. Mean values with standard deviations of three independent biological replicates are depicted in the graph. Significance testing was performed using multiple unpaired t-tests with Holm-Šídák method, and significance levels are indicated on the graphs: * p < 0.05 and ns: not significant. Fold changes of promoter activity between *L. rhamnosus* GR-1 and *L. rhamnosus* GG are also depicted on the graph.


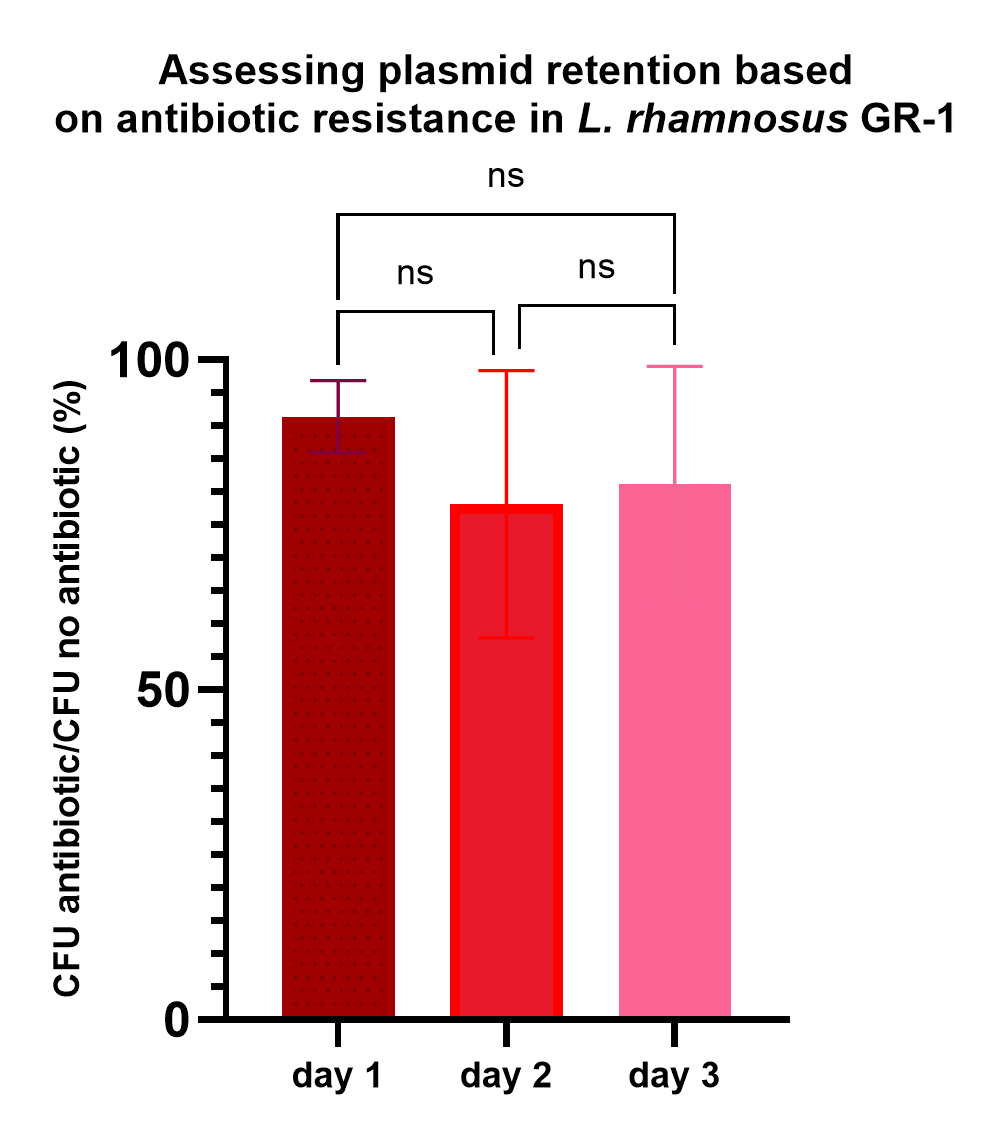


**Figure S2. Assessing plasmid retention based on antibiotic resistance of pAMB6500 in *L. rhamnosus* GR-1.** Overnight cultures of *L. rhamnosus* GR-1 containing pAMB6500 were sub cultured every 24 hours in media without antibiotics. Cultures were plated in serial dilutions on plates containing antibiotics and plates without antibiotics. Colony Forming Units (CFUs) were counted and ratio of CFUs in selective conditions against CFUs in non-selective conditions was plotted in percentages on the y-axis. This experiment was conducted on three consecutive days. Mean values with standard deviations of three independent biological replicates are depicted in the graph. Significance testing was performed using one-way ANOVA with Tukey correction but no significant (ns) differences between conditions could be detected.


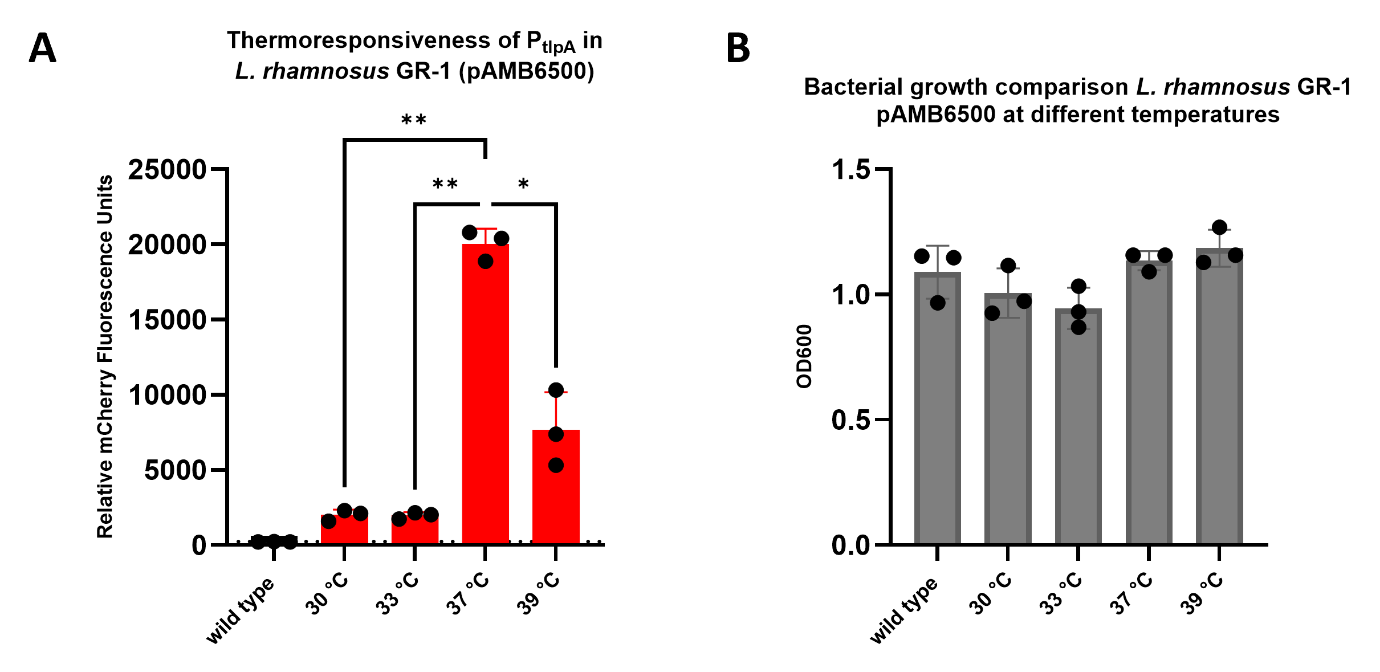


**Figure S3.** **Native thermo-responsiveness of P_tlpA_ in *L. rhamnosus* GR-1.** Recombinant *L. rhamnosus* GR-1 containing the pAMB6500 plasmid was grown at different temperatures (30°C, 33°C, 37°C, and 39°C), after which mCherry fluorescence was measured (A). Fluorescence values were corrected for bacterial growth, measured as OD_600_ and shown on the figure as ‘Relative mCherry fluorescence units’. Mean values with standard deviations of three independent biological replicates are depicted in the graph. Significance testing was performed against *L. rhamnosus* GR-1 pAMB6500 grown at 37°C using Welch’s ANOVA with Dunnett T3 correction. Significance levels (* p-value < 0.05, ** p-value < 0.005) are indicated on the graph. No significant differences in OD_600_ of overnight cultures grown at different temperatures could be observed (B).


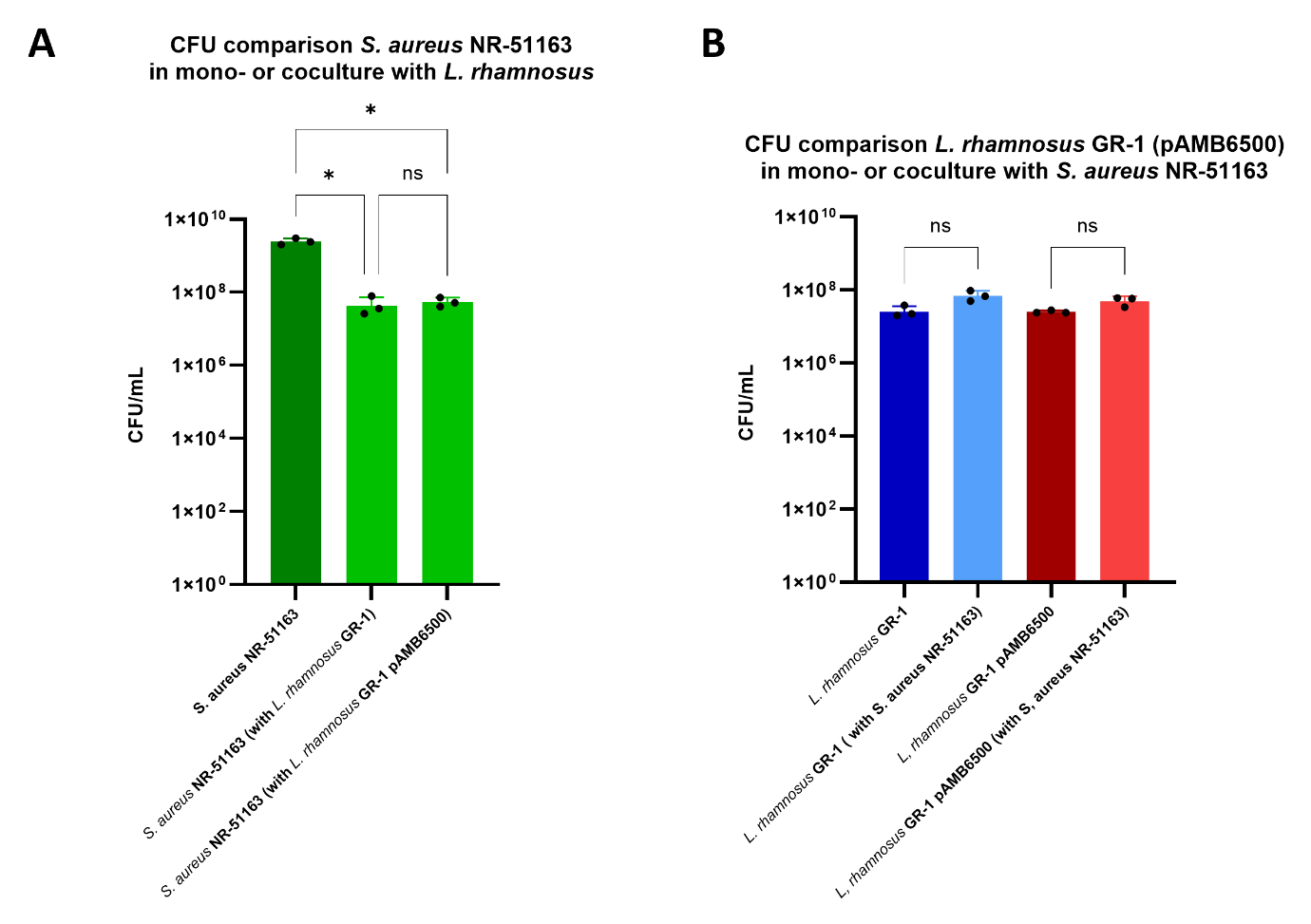


**Figure S4. Colony Forming Units (CFU) comparison of *S. aureus* NR-51160, *L. rhamnosus* GR-1 and *L. rhamnosus* GR-1 pAMB6500 grown in single and combination cultures.** A) Comparison of bacterial growth *S. aureus* NR-51160 grown as single culture compared to when grown combined with *L. rhamnosus* GR-1 or *L. rhamnosus* GR-1 pAMB6500. Significant growth reduction can be observed for *S. aureus* NR-51163 when combined with *L. rhamnosus* GR-1 or *L. rhamnosus* GR-1 pAMB6500. B) Comparison of *L. rhamnosus* GR-1 and *L. rhamnosus* GR-1 pAMB6500 grown as a single culture compared to when grown in combination with *S. aureus* NR-51163. No significant growth difference could be observed between single and combination culture conditions. Combination cultures were spotted in serial dilution on selective agar containing BHI with 10 µg/mL trimethoprim for *S. aureus* NR-51163, MRS with 10 µg/mL erythromycin for *L. rhamnosus* GR-1 pAMB6500. For wild type *L. rhamnosus* GR-1, MRS agar plates without antibiotics were used. Mean values with standard deviations of three independent biological replicates are depicted in the graph. Significance testing was performed using Welch’s ANOVA with Dunnett T3 correction and significance levels are indicated on the graphs: * p < 0.05 and ns: not significant.


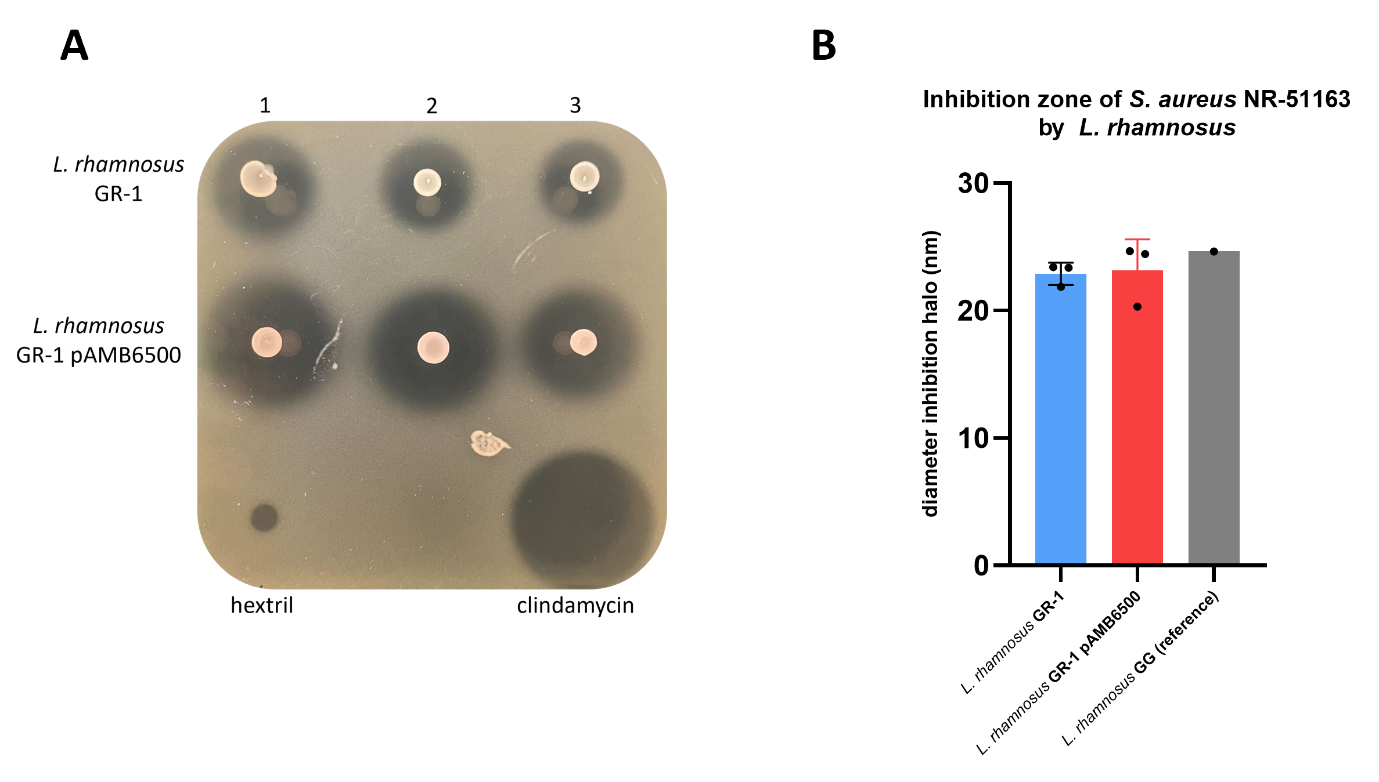


**Figure S5. Bacterial spot assays investigating inhibitory effect of *L. rhamnosus* GR-1 and *L. rhamnosus* GR-1 pAMB6500 on the growth of *S. aureus* NR-51163.** Spots of overnight cultures of *L. rhamnosus* GR-1 (wild type and pAMB6500) were added to the plate and incubated at 37°C for 48 hours. After that, soft agar top layer containing *S. aureus* NR-51163 was added to the plate. After 24 hours incubation, the plate was visualised showing clear halos indicating inhibition of *S. aureus* growth by *L. rhamnosus* strains (A). Hextril and clindamycin were included as positive controls. B) Diameter of inhibition zones (halos) in nanometer (nm) were measured and plotted. Three independent biological replicates were included.
